# Development of the breastfed infant oral microbiome over the first two years of life in the BLOSOM Cohort

**DOI:** 10.3389/fcimb.2025.1534750

**Published:** 2025-04-15

**Authors:** Roaa A. Arishi, Ali S. Cheema, Ching T. Lai, Matthew S. Payne, Donna T. Geddes, Lisa F. Stinson

**Affiliations:** ^1^ School of Molecular Sciences, The University of Western Australia, Crawley, WA, Australia; ^2^ Australian Breastfeeding + Lactation Research and Science Translation (ABREAST) Network, Perth, WA, Australia; ^3^ The University of Western Australia (UWA) Centre for Human Lactation Research and Translation, Crawley, WA, Australia; ^4^ Ministry of Education, Riyadh, Saudi Arabia; ^5^ The Kids Research Institute Australia, Nedlands, WA, Australia; ^6^ Division of Obstetrics and Gynaecology, The University of Western Australia, Crawley, WA, Australia

**Keywords:** oral microbiome, infant diet, breastfeeding, human milk, infancy

## Abstract

**Background:**

Acquisition and development of the oral microbiome are dynamic processes that occur over early life. This study aimed to characterize the temporal development of the oral microbiome of predominantly breastfed infants during the first two years of life.

**Methods:**

Infant oral samples (n=667) were collected at ten time points from the first week to year two of life from 84 infants. Bacterial DNA profiles were analyzed using full-length 16S rRNA gene sequencing.

**Results:**

The oral microbiome was dominated by *Streptococcus mitis*, *Gemella haemolysans*, and *Rothia mucilaginosa*. Bacterial richness decreased from 1 to 2 months (P = 0.043) and increased from 12 to 24 months (P = 0.038). Shannon diversity increased from 1 week to 1 month and again from 6 to 9 months and 9 to 12 months (all P ≤ 0.04). The composition of the infant oral microbiome was associated with multiple factors, including early pacifier use, intrapartum antibiotic prophylaxis, maternal allergy, pre-pregnancy body mass index, siblings, delivery mode, maternal age, pets at home, and birth season (all P < 0.01). Introduction of solid foods was a significant milestone in oral microbiome development, triggering an increase in bacterial diversity (richness P = 0.0004; Shannon diversity P = 0.0007), a shift in the abundance of seven species, and a change in beta diversity (P = 0.001).

**Conclusion:**

These findings underscore the importance of multiple factors, particularly the introduction of solid foods, in shaping the oral microbiome of breastfed infants during early life.

## 1 Introduction

The oral microbiome plays an essential role in both oral and systemic health (Jia et al., 2018; Nardi et al., 2021). In the first two years of life, the oral microbiome undergoes significant changes as infants progress from a predominantly milk-based diet to the introduction of solid foods (Oba et al., 2020). This window of time is characterized by rapid microbial succession and is influenced by various factors. Current evidence suggests that, the infant oral microbiome is predominantly composed of *Streptococcus* (Drell et al., 2017; Verma et al., 2018) with *Gemella*, *Rothia*, *Haemophilus*, and *Lactobacillus* also prevalent (Blum et al., 2022). However, the temporal development of the oral microbiome during early life and the influence of various maternal, infant, and environmental covariates remains underexplored. Previous studies have highlighted that pacifier use (Plonka et al., 2012), intrapartum antibiotic prophylaxis (Gomez-Arango et al., 2017; Li et al., 2019), and mode of delivery (Li et al., 2005, 2018; Holgerson et al., 2011; Nelun Barfod et al., 2011; Thakur et al., 2012; Dominguez-Bello et al., 2016; Hurley et al., 2019) are associated with the composition and diversity of the infant oral microbiome. However, comprehensive longitudinal data capturing these influences over the early years of life are limited.

Solid food introduction also represents a crucial milestone during infancy, influencing microbial colonization and diversity in the oral cavity with demonstrated shifts in oral microbiome composition (Sulyanto et al., 2019; Oba et al., 2020). For instance, one study of nine infants reported an increase in bacterial diversity and richness and decreased relative abundance of *Streptococcus mitis* with food introduction (Sulyanto et al., 2019). Another study of 12 infants found a rise in the relative abundance of *Gemella*, *Neisseria*, *Veillonella*, and *Fusobacterium* following solid food introduction (Oba et al., 2020). Unfortunately, both studies have limitations related to small sample sizes and infrequent sampling. The small sample sizes of these studies were further complicated by the inclusion of a mix of breastfed, mixed fed, and formula fed infants. Given the significant impact of breastfeeding on the oral microbiome (Sulyanto et al., 2019; Oba et al., 2020), different milk-feeding styles should not be grouped for such an analysis.

Cessation of breastfeeding may represent another inflection point in infant oral microbiome development; however, no studies have investigated this. Nevertheless, infants partially breastfed for at least 12 months have been shown to harbor different oral microbiota compared to those who ceased breastfeeding by 12 months (n=90) (Dzidic et al., 2018). These differences persisted up to 7 years of age, with the group that continued breastfeeding showing higher relative abundance of *Streptococcus* at 12 months and *Veillonella* at 7 years of age. However, distinguishing the effects of breastfeeding cessation from those of formula supplementation remains challenging.

The aim of this study was to comprehensively characterize the temporal development of the oral microbiome in exclusively and predominantly breastfed infants over the first two years of life. In addition, we aimed to identify maternal, infant, and environmental determinants of early oral microbiome development.

## 2 Materials and methods

### 2.1 Study design

Pregnant women were recruited to participate in the BLOSOM cohort (Breastfeeding Longitudinal Observational Study of Mothers and kids), a prospective birth cohort that was recruited in Perth (Western Australia). A total of 84 women were enrolled during the third trimester of pregnancy. Non-smoking women with singleton pregnancies and no significant health conditions or pregnancy complications who intended to breastfeed for at least 12 months and went on to deliver full term infants were invited to participate. Participants provided informed written consent. The study was approved by the Human Research Ethics Committee at The University of Western Australia (RA/4/20/4023) and participants gave written informed consent.

### 2.2 Sample and data collection

Mothers completed a background questionnaire at the time of enrolment to ascertain their health history and demographic metadata. Another questionnaire was administered within the first week following birth to obtain data on birthing experiences and early feeding. At each sample collection time point mothers completed a questionnaire regarding their infant feeding practices, their own health and medications, and those of their infant. The average volume of formula consumed by those infants who were supplemented with formula in this cohort (n=30) was 100 ml/day. Samples were collected at ten time points across the first 2 years of life: 2– 7 days, 1, 2, 3, 4, 5, 6, 9, 12, and 24 months postpartum.

Mothers were provided with written instructions for collecting their infant’s oral samples. Infant oral samples were collected with COPAN E-swabs, which were firmly rubbed up and down and in a circular motion against the interior of the cheek ten times. This procedure was then repeated on the contralateral cheek using the same swab. Swabs were removed from the mouth without contacting other surfaces and stored in the participants home refrigerator for up to 18 hours before being transported on ice to the laboratory, where they were immediately processed. Oral swabs were eluted into the collection media (Liquid Amies) by vortexing for 5 seconds, then aliquoted into sterile tubes and stored at -80°C until subsequent analysis.

### 2.3 DNA extraction

Swab media was centrifuged at 40,000 × *g* for 5 min at 4°C, and the supernatant was discarded. DNA extraction from the cell pellet was carried out using the QIAGEN MagAttract Microbial DNA Isolation Kit according to the manufacturer’s instructions. A negative extraction control (reagents only) was included in each batch.

### 2.4 16S rRNA gene amplification and sequencing

The full-length 16S rRNA gene was amplified using asymmetrically barcoded primers 27F and 1492R. PCR was carried out in 30 µL reactions containing 0.75 µL each of DTT and dsDNase (ArcticZymes PCR decontamination kit), 0.3 μM each of the forward and reverse barcoded primers, 1X AccuStart II ToughMix, 3 µL nuclease-free water, and 6 µL of template. All master mix reagents were treated with the ArcticZymes PCR Decontamination kit prior to addition of template. One no template control was included in each batch. The PCR cycling conditions consisted of an initial heating step for 3 minutes at 94°C, followed by 35 cycles of 94°C for 30 seconds, 52°C for 30 seconds, and 72°C for 2 minutes, with a final extension step at 72°C for 5 minutes. To confirm the presence and size of amplicons, PCR products were visualized on a QIAxcel capillary gel electrophoresis system using a DNA High-Resolution cartridge. Barcoded amplicons were normalized and multiplexed into pools, which underwent purification and concentration using Macherey-Nagel NucleoMag NGS beads. Purified amplicon pools were sequenced on a PacBio Sequel II at the Australian Genome Research Facility (AGRF).

### 2.5 Sequencing data processing

Full-length 16S rRNA gene sequence data were processed using Mothur v.1.48.0 (Schloss et al., 2009). Raw sequence data underwent length (1336-1743 bp) and homopolymer (≤9) filtering. Sequences were aligned to the SILVA reference alignment v132 (Quast et al., 2013) and VSEARCH was used to eliminate chimeric sequences (Rognes et al., 2016). Sequences were initially classified using the SILVA taxonomy database (v132) (Quast et al., 2013), and a confidence threshold of 80. Non-bacterial sequences were excluded. Subsequently, sequences were clustered into operational taxonomic units (OTUs).

OTUs with an average relative abundance of >0.5% (n=18) were classified using BLAST (Altschul et al., 1997) with a cutoff of 97% sequence identity and 99% sequence coverage. Sequences recovered from the negative extraction and template controls are reported in **Supplementary Table S1**.

### 2.6 Data analysis

Diversity analyses were performed on subsampled data. Subsampling was performed at 2096 reads, resulting in an average coverage of 85.6% and exclusion of 13 low-yield samples. Alpha diversity was assessed by OTU-level richness and Shannon diversity. Beta diversity was assessed by Bray-Curtis dissimilarity. Differential abundance analyses were performed on center log ratio (CLR)-transformed OTU data (Fry and Aitchison, 1987). All statistical analyses were performed in R (Xia and Sun, 2023).

Longitudinal changes in oral bacteria were analyzed using linear mixed effects models with the MaAsLin2 R package (Mallick et al., 2021). 1 month was used as the reference time point and infant ID was included as a random effect. To assess changes in alpha diversity between subsequent time points, Kruskal-Wallis rank sum tests were performed with pairwise Wilcoxon rank sum tests with continuity correction. Changes in infant oral microbiome community structure over time were assessed using PERMANOVA with the adonis2 function of the R vegan package with 999 permutations (Dixon, 2003), stratified by infant ID to account for repeated measures. Differences in beta diversity between subsequent time points were measured using pairwise PERMANOVA.

The influence of solid food introduction was assessed by comparing samples collected immediately before and at two subsequent time points after the initial intake of solid foods. We performed Kruskal-Wallis and Wilcoxon rank-sum tests to identify changes in the bacterial composition and alpha diversity metrics. The impact of introduction of solid foods on beta diversity was assessed by PERMANOVA using the adonis2 function of the R vegan package with 999 permutations.

The impact of cessation of breastfeeding was assessed by comparing oral microbiome composition between weaned and breastfed infants at 24 months of age. This analysis was not achievable at earlier time points due to the high rates of breastfeeding in this cohort (all infants received breast milk until at least 9 months of age, and only two had ceased breastfeeding by the 12-month time point). Wilcoxon rank sum tests were used to assess differences in alpha diversity metrics and CLR-transformed OTU abundances. PERMANOVA was used to assess differences in beta diversity between groups.

To investigate the influence of maternal, infant, and environmental factors on the infant oral microbiome, we employed multivariate linear mixed effects models using the lme4 R package and AIC model selection. Models were fitted for each of the 18 OTUs tested here that had an average abundance >0.5%, in addition to Shannon diversity and richness, with the following explanatory variables: maternal age at delivery, maternal pre-pregnancy BMI category (underweight/normal weight or overweight/obese), maternal allergy, intrapartum antibiotic exposure, birth season (spring/summer or autumn/winter), delivery mode (vaginal, planned (pre-labor) caesarean section, or intrapartum caesarean section), presence of siblings in the home, presence of furry pets (cats or dogs) in the home, and pacifier use within the first week of life. Birth season was divided into two categories (spring/summer or autumn/winter) due to the low number of infants born in summer (n=7). Similarly, underweight/normal weight pre-pregnancy BMI and overweight/obese pre-pregnancy BMI were grouped together due to a low number of mothers with an underweight or mothers with obesity (n=3 and n=7 respectively). Infant age was included as an interaction term for each variable, and infant ID was included as a random effect. 1 month was used as the reference time point. The influence of these maternal, infant, and environmental factors on infant oral beta diversity was modelled using adonis2 in the vegan package (Dixon, 2003) using backwards model selection with 999 permutations. Infant age was included as an interaction for each explanatory variable and the analysis was stratified by infant ID.

## 3 Results

**Figure 1:**
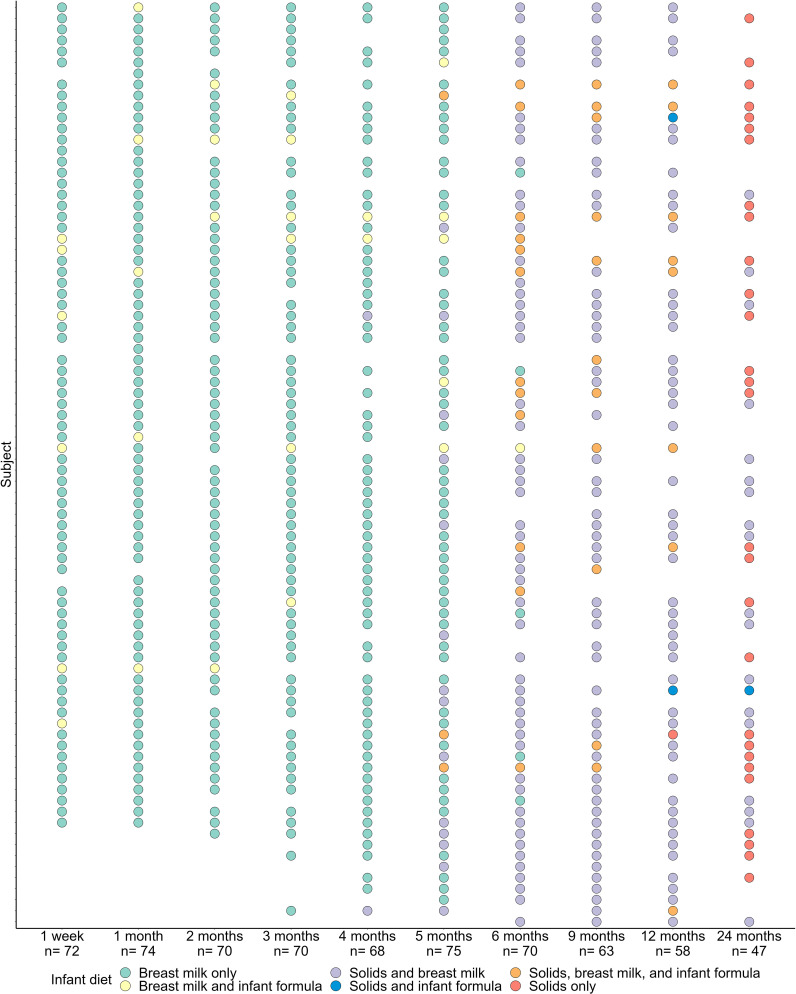
Sample collection and infant feeding mode at each of the 10 sample collection time points over the first two years of life.

To investigate the temporal development of the oral microbiome during early life, we performed full-length 16S rRNA gene sequencing on 667 oral samples collected from 84 infants in the BLOSOM cohort. Maternal and infant characteristics are detailed in **Table 1**. All infants were born at term without major health complications and were exclusively or predominantly breastfed prior to the introduction of solid foods. Samples were collected at ten time points across the first two years of life (**Figure 1**).

**Table 1:** Maternal and infant characteristics (n=84).

Characteristic	Mean (range) or n (%)
Maternal age at delivery (years)	32.8 (25.0-46.4)
Maternal ethnicity	
Caucasian	74 (88.1%)
Other	10 (11.9%)
Delivery mode	
Vaginal	58 (69.1%)
Elective caesarean	16 (19.1%)
Non-elective caesarean	10 (11.9%)
Intrapartum antibiotic exposure	
Yes	35 (44.3%)
Maternal pre-pregnancy BMI (kg/m^2^)	24.0 (15.6-43.7)
Underweight (< 18.5)	3 (4.8%)
Normal (18.5–24.9)	41 (66.1%)
High (Overweight) (25.0–29.9)	11 (17.7%)
Obese (≥ 30)	7 (11.2%)
Maternal allergies (self-reported)	
Yes	27 (32.1%)
Birth season	
Spring	28 (33.3%)
Summer	7 (8.3%)
Autumn	24 (28.5%)
Winter	25 (29.7%)
Infant sex	
Female	44 (52.3%)
Pacifier use within the first week	
Yes	18 (22.7%)
Age of first solid food intake (weeks)	23.3 (16-39)
Siblings in the home	
Yes	64 (76.1%)
Furry pets in the home	
Yes	48 (75.0%)

### 3.1 Evolution of the oral microbiome over the first two years of life

**Figure 2:**
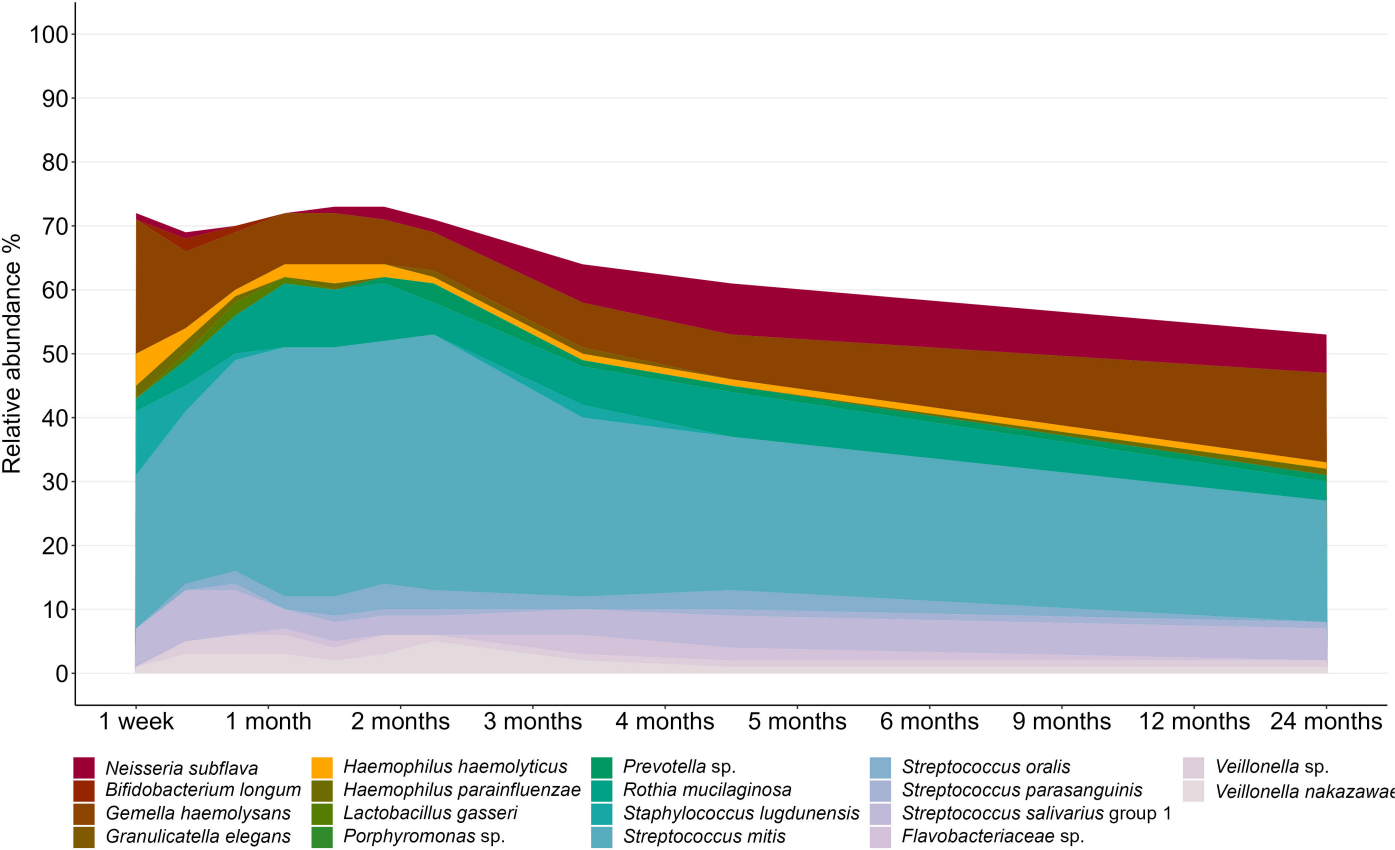
Composition of the infant oral microbiome over the first two years of life. OTUs with an average relative abundance of >0.5% are displayed.

**Figure 3:**
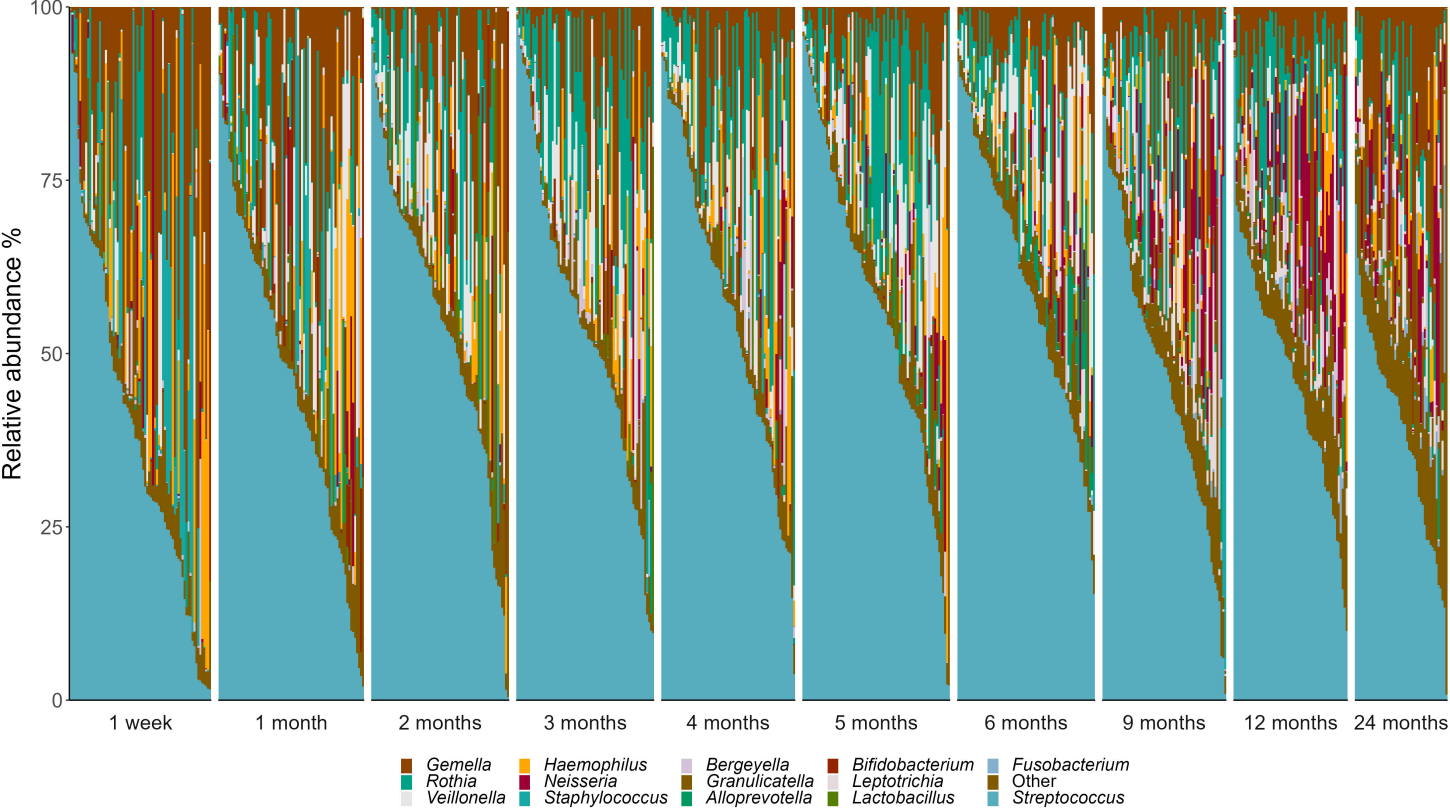
Genus-level interindividual variation in the infant oral microbiome. Individuals are represented by vertical bars. Genera that made up <2% relative abundance at all time points are grouped together as “Others”.

Within this predominantly breastfed cohort, *Streptococcus* emerged as the dominant bacterial genus, accounting for 50.8% of infant oral microbiota profiles. At the OTU level, *S. mitis* (31.9%), *Gemella haemolysans* (10.4%), and *Rothia mucilaginosa* (7.4%) were the most abundant taxa (**Figure 2**). However, there was a high level of interindividual variation, with some samples made up almost exclusively by *Streptococcus* spp. while others had very little of this genus (0.04-97.4%; **Figure 3**).

**Figure 4:**
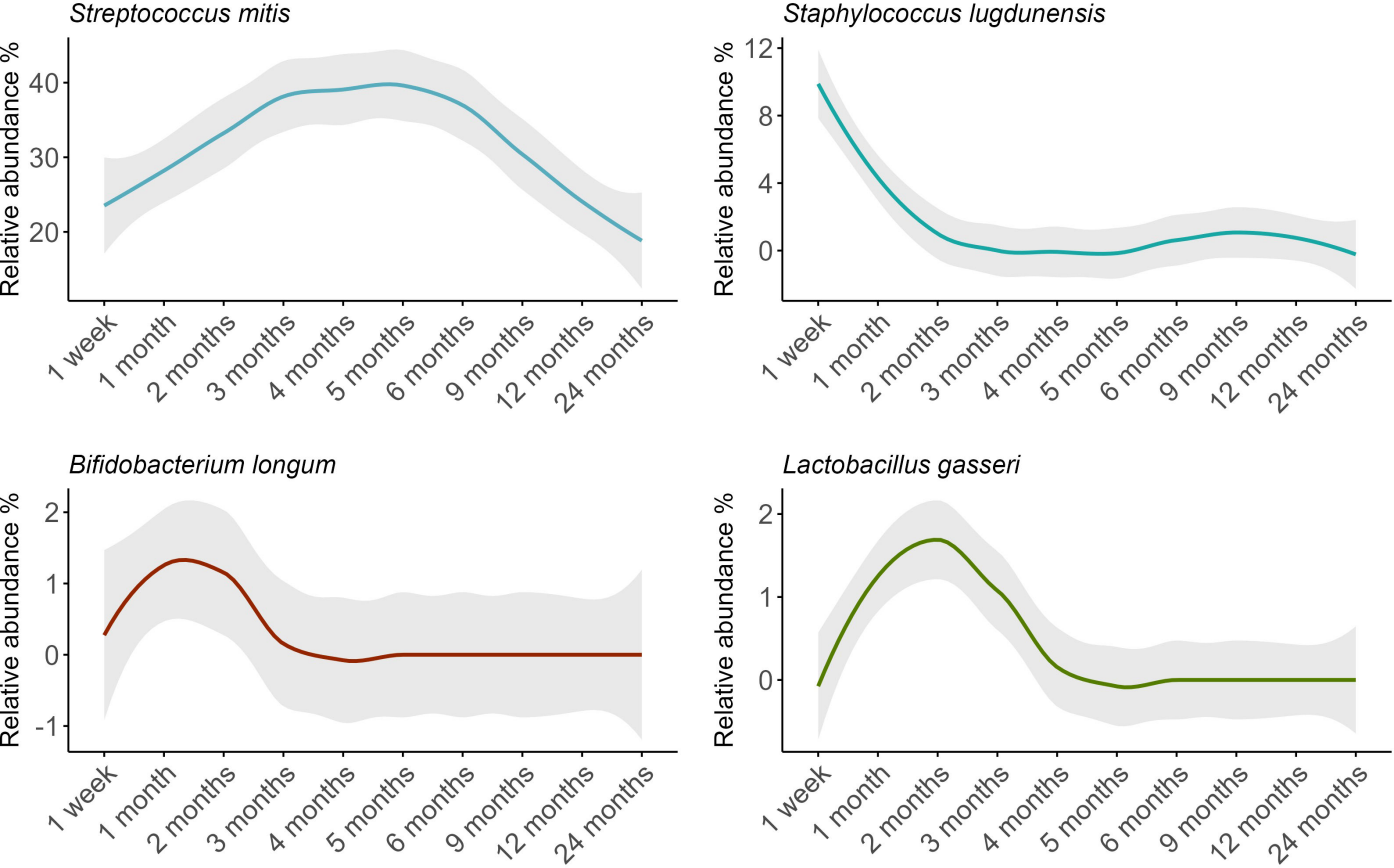
Relative abundance trends of key bacterial species in infant oral microbiomes over time, with loess-smoothed lines. The shaded area represents the 95% confidence intervals.

The composition of the oral microbiome was dynamic over time, with fluctuations in 17 of the 18 OTUs analyzed here (**Figure 4**; **Supplementary Table S2**). Compared to samples taken at 1 month, *S. mitis* showed a higher relative abundance at 3 months and lower relative abundances at 12 and 24 months (P ≤ 0.02). *Staphylococcus lugdunensis* showed a higher relative abundance at 1 week, then lower relative abundance for all time points from 2 months to 24 months (P ≤ 0.006). Interestingly, the lactic acid bacteria *Bifidobacterium longum* and *Lactobacillus gasseri* both peaked at 1-2 months, before rapidly declining in abundance (*B. longum* 4 to 24 months P ≤ 0.007; *L. gasseri* 4 months to 24 months P **<** 0.001.

**Figure 5:**
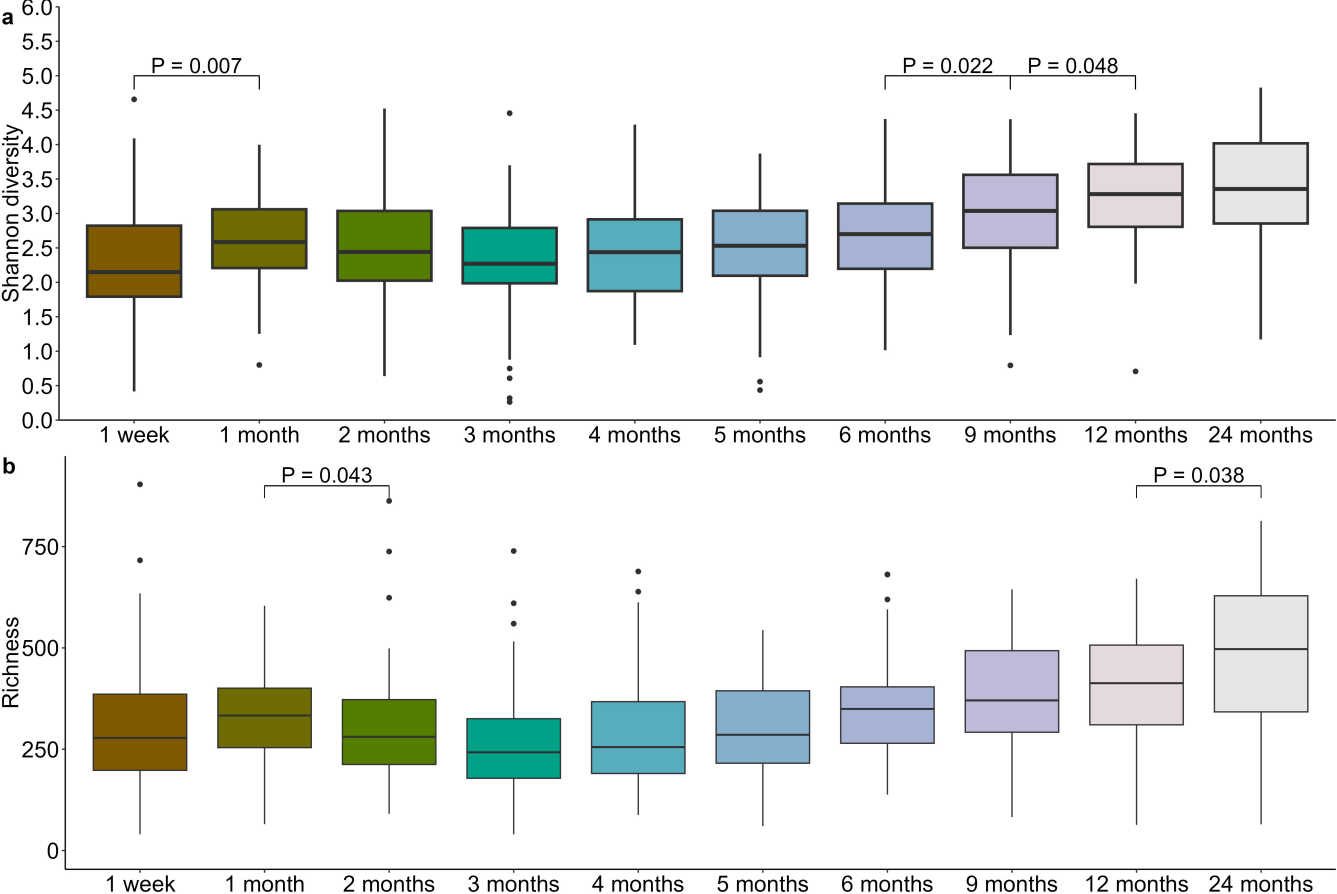
Infant oral microbiome evenness **(a)** and richness **(b)** increases after the first 6 months of life.

Diversity of the infant oral microbiome varied significantly over the first two years of life (richness and Shannon diversity P < 0.0001). Bacterial richness decreased significantly from 1 month to 2 months (P = 0.043), before rising again with a significant increase observed from 12 months to 24 months (P = 0.038) (**Figure 5a**; **Supplementary Table S3**). Shannon diversity underwent an initial increase from 1 week to 1 month (P = 0.007) before again increasing from 6 to 9 months (P = 0.022) and 9 to 12 months (P = 0.048) (**Figure 5b**; **Supplementary Table S3**).

**Figure 6:**
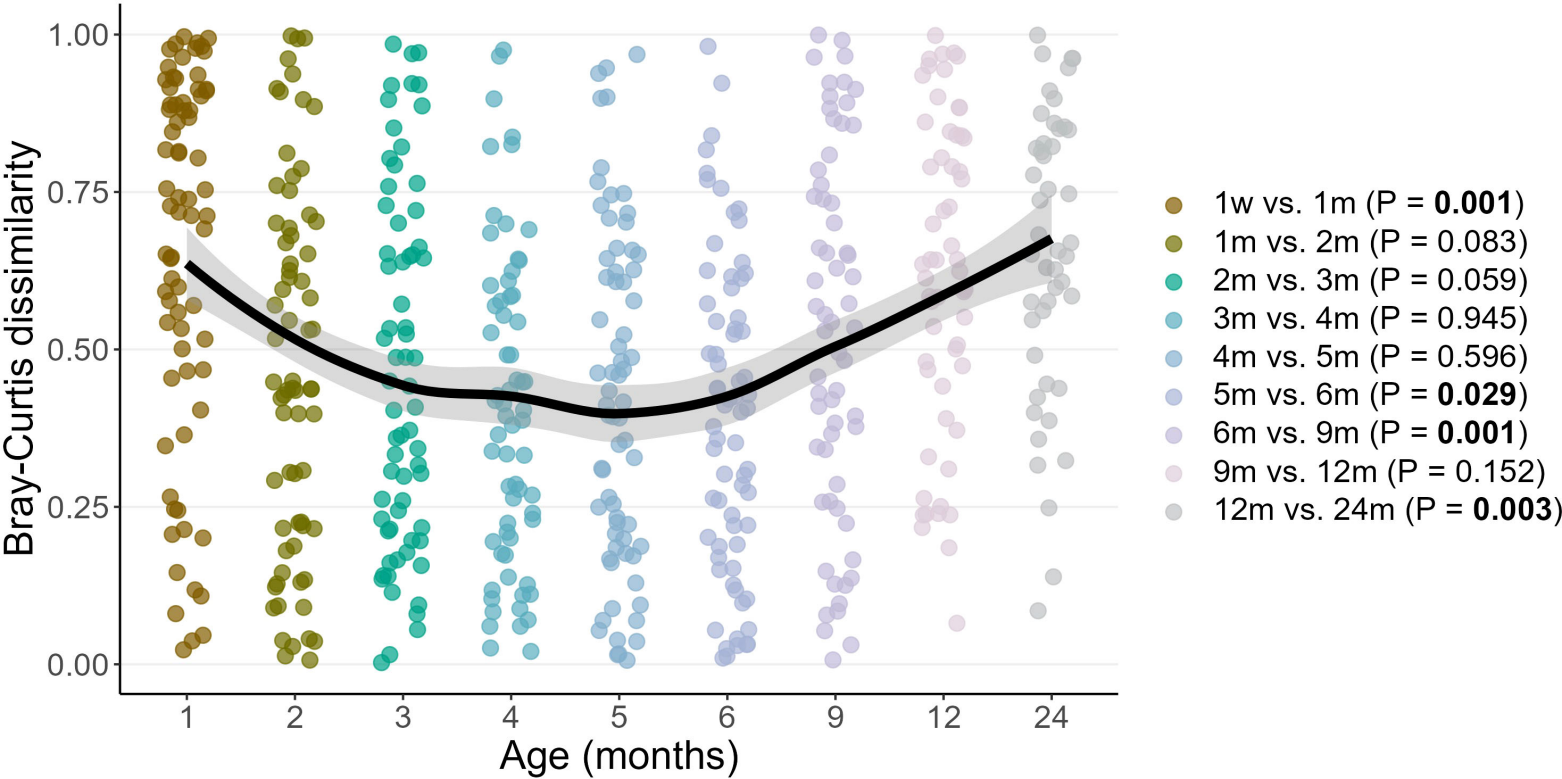
Pairwise Bray–Curtis dissimilarities between subsequent time points over two years of life. Each point represents the Bray-Curtis distance between samples at consecutive time points for an individual infant. Age on the X axis represents the latter sample time point in the comparison. Loess lines are fitted to the data with shaded areas representing the 95% confidence intervals.

Infant oral microbiome development progressed significantly over time (PERMANOVA R^2^ = 0.081, P = 0.001). We observed an initial dynamic phase of establishment, as illustrated by the significant Bray-Curtis dissimilarity between the 1 week and 1 month samples (PERMANOVA R^2^ = 0.023, P = 0.001). The oral microbiome then entered a stable phase from 2-5 months of age, corresponding with the exclusive/predominant breastfeeding period. Community structure became dynamic again from 6 months of age onwards (5 vs. 6 months PERMANOVA, R^2^ = 0.014, P = 0.029; 6 vs. 9 months PERMANOVA, R^2^ = 0.031, P = 0.001; 12 vs. 24 months PERMANOVA, R^2^ = 0.031, P = 0.003), (**Figure 6**).

### 3.2 Determinants of the infant oral microbiome

**Figure 7:**
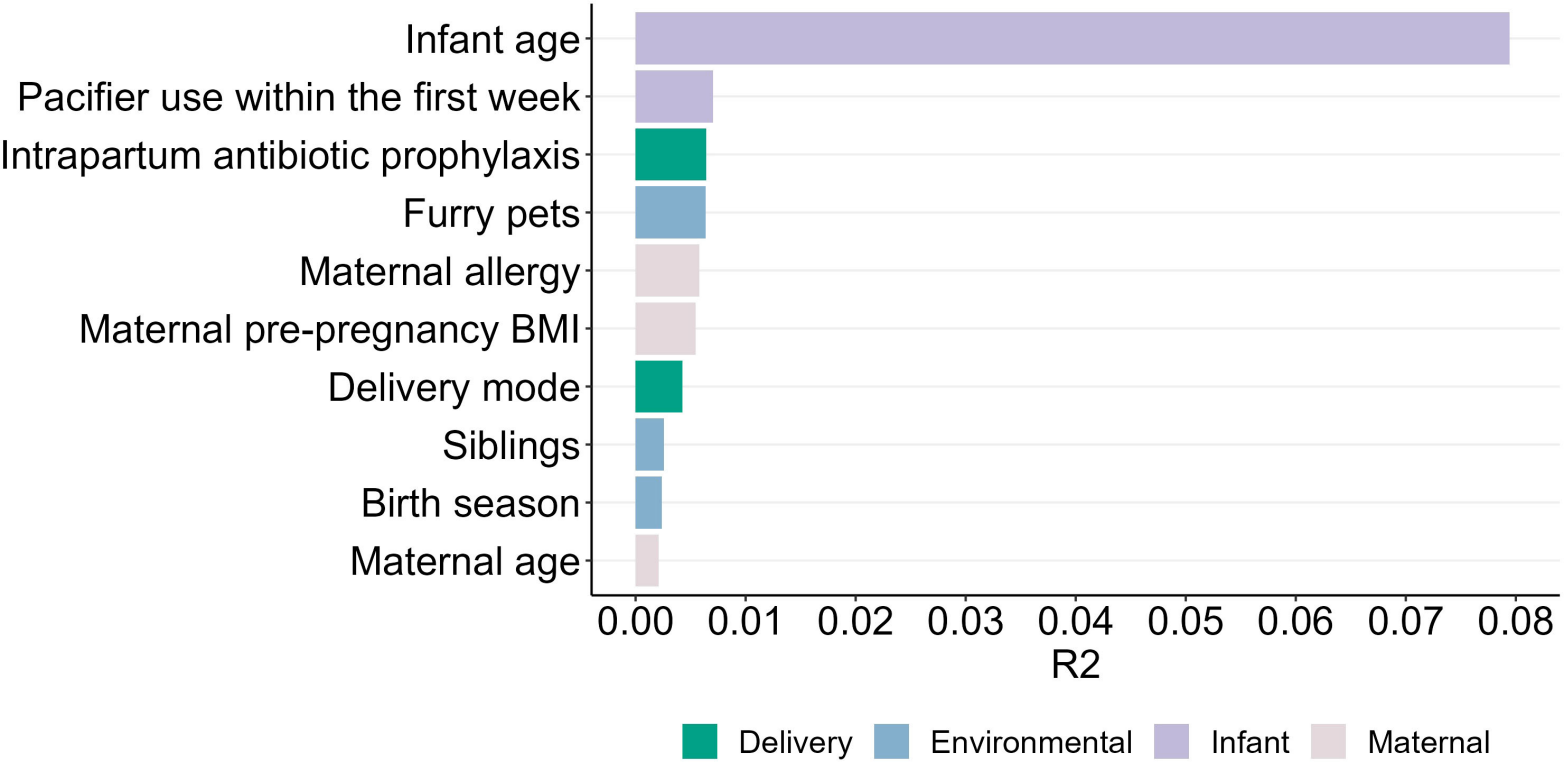
Explained variance (R²) of factors influencing the oral microbiome.

Numerous significant associations were identified between maternal, infant, and environmental factors and the infant oral microbiome (**Figure 7**; **Supplementary Table S4**).

#### 3.2.1 Maternal determinants

**Figure 8:**
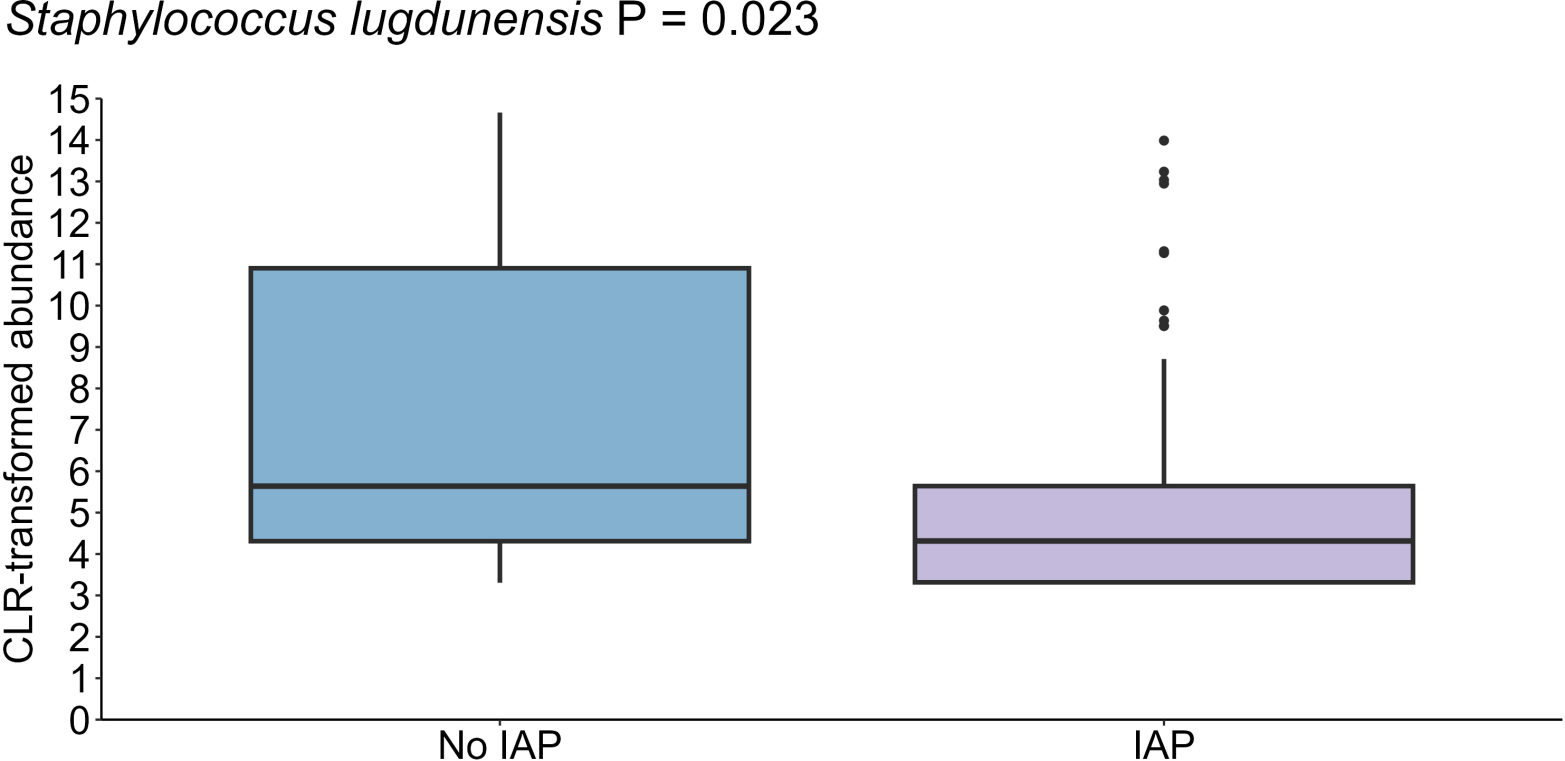
Reduced levels of *S. lugdunensis* in the oral cavity of infants born to mothers who received antibiotics during delivery (n = 35) compared to those born to mothers who did not receive antibiotics (n = 44) (P = 0.023). Data shown for the 1-month time point, with 1 month as the reference for age comparisons.

Maternal intrapartum antibiotic prophylaxis explained a small but significant difference in the infant oral microbiome (PERMANOVA R^2^ = 0.005, P = 0.001) (**Supplementary Figure S1**). In this cohort, 33.0% of intrapartum antibiotics were administered for caesarean section (typically cefazolin), and 5.1% for Group B Streptococcus colonization or prolonged rupture of membranes (typically penicillin). The relative abundance of *S. lugdunensis*, a species that is largely susceptible to both of these antibiotics, was significantly lower in the oral microbiome of infants of antibiotic exposed mothers (P = 0.023) (**Figure 8**). Shannon diversity and richness were not different based on intrapartum antibiotic prophylaxis.

Delivery mode was also significantly associated with the infant oral microbiome (PERMANOVA R^2^ = 0.004, P = 0.001) (**Supplementary Figure S2**). Infants delivered via planned caesarean section exhibited lower oral richness (P = 0.029) and Shannon diversity (P = 0.026) compared to those delivered vaginally (**Supplementary Figure S3**).

Maternal pre-pregnancy BMI was significantly associated with the infant oral microbiome (PERMANOVA R^2^ = 0.005, P = 0.001) (**Supplementary Figure S4**). Infants born to mothers with OW/OB BMI demonstrated higher abundances of *Neisseria subflava* (P = 0.016) and *Prevotella* sp. (P = 0.002) compared to those born to mothers (**Supplementary Figure S5**) with a normal or underweight BMI. No difference was observed in Shannon diversity and richness.

Increased maternal age (PERMANOVA R^2^ = 0.002, P = 0.001) and allergy status (PERMANOVA R^2^ = 0.005, P = 0.001) were also significant covariates for the infant oral microbiome (**Supplementary Figures S6, 7**); however, they were not associated with alpha diversity nor the relative abundance of any individual OTU.

#### 3.2.2 Infant determinants

**Figure 9:**
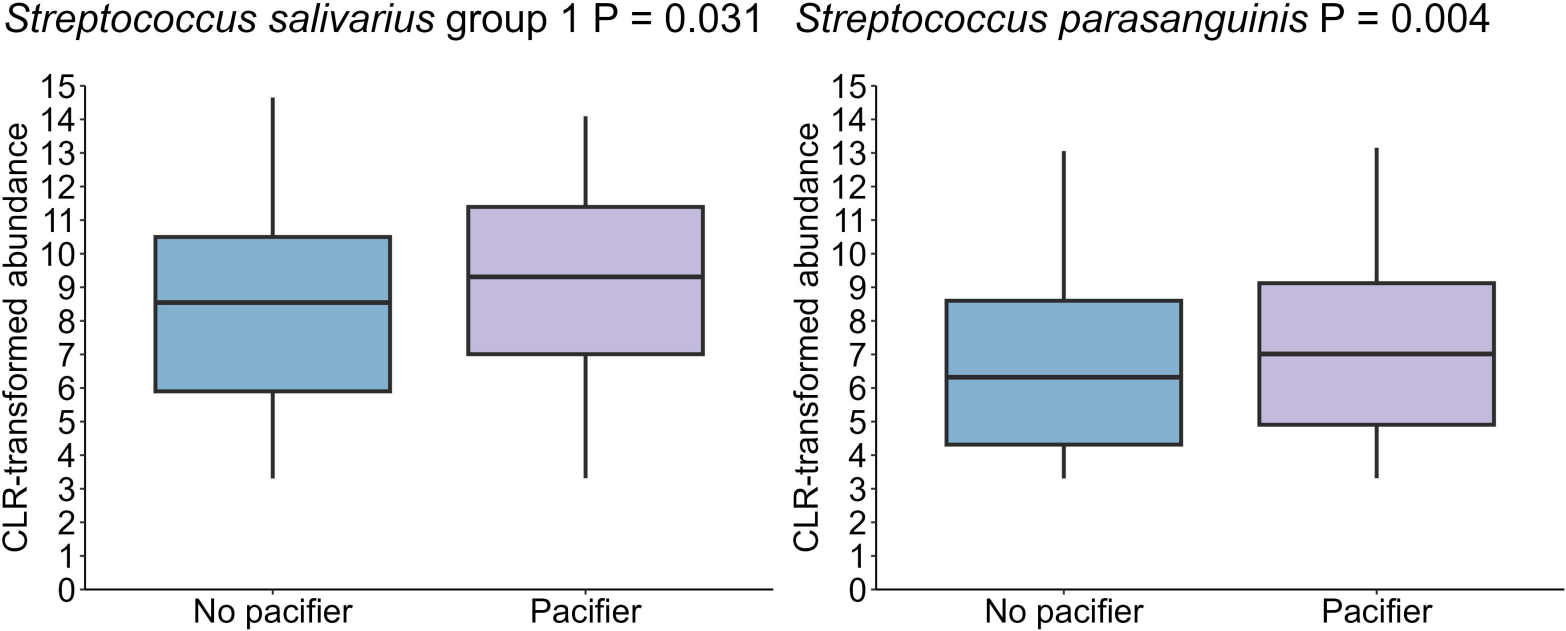
A higher levels of *S. salivarius* group 1 and *S. parasanguinis* in the oral cavity of infants who used a pacifier in the first week post-partum (n = 18) compared to those who did not use pacifiers (n = 61) (P =0.031, P = 0.004, respectively). Data shown for the 1-month time point, with 1 month as the reference for age comparisons.

Of the microbiome covariates tested here, pacifier use in the first week of life had the greatest, albeit still minor, effect on the oral microbiome after infant age (PERMANOVA R^2^ = 0.007, P = 0.001) (**Supplementary Figure S8**). Infants who used pacifiers exhibited a higher abundance of *Streptococcus salivarius* group 1 (P = 0.031), and *Streptococcus parasanguinis* (P = 0.004) than those who did not use pacifiers (**Figure 9**). However, Shannon diversity and richness were not different relative to pacifier use.

#### 3.2.3 Environmental determinants

The presence of dogs or cats in the household explained a small but significant amount of variance in the infant oral microbiome (PERMANOVA R^2^ = 0.006, P = 0.001) (**Supplementary Figure S9**). Infants residing in homes with furry pets exhibited a lower abundance of *Prevotella* sp. (P = 0.018) compared to those without pets (**Supplementary Figure S10**) with no differences in Shannon diversity and richness. Similarly, the presence of older siblings in the household was significantly associated with infant oral microbiome composition (PERMANOVA R^2^ = 0.008, P = 0.001) (**Supplementary Figure S11**). However, no significant differences were detected in the relative abundance of the most abundant bacterial species.

Birth season explained minor variations in the infant oral microbiome (PERMANOVA R^2^ = 0.002, P = 0.001) (**Supplementary Figure S12**). Infants born during autumn/winter exhibited a lower abundance of *S. parasanguinis* (P = 0.007) compared to those born in summer/spring, with no statistically significant difference observed in Shannon diversity and richness between the two groups (**Supplementary Figure S13**).

### 3.3 Introduction of solid foods impacts the oral microbiome to a far greater extent than weaning

Infants began consuming solid foods at varying ages, with approximately 76.1% initiating solids by the age of 6 months. 20.2% of infants started solids by 5 months, while a minority began as early as 4 months (2.4%).

**Figure 10:**
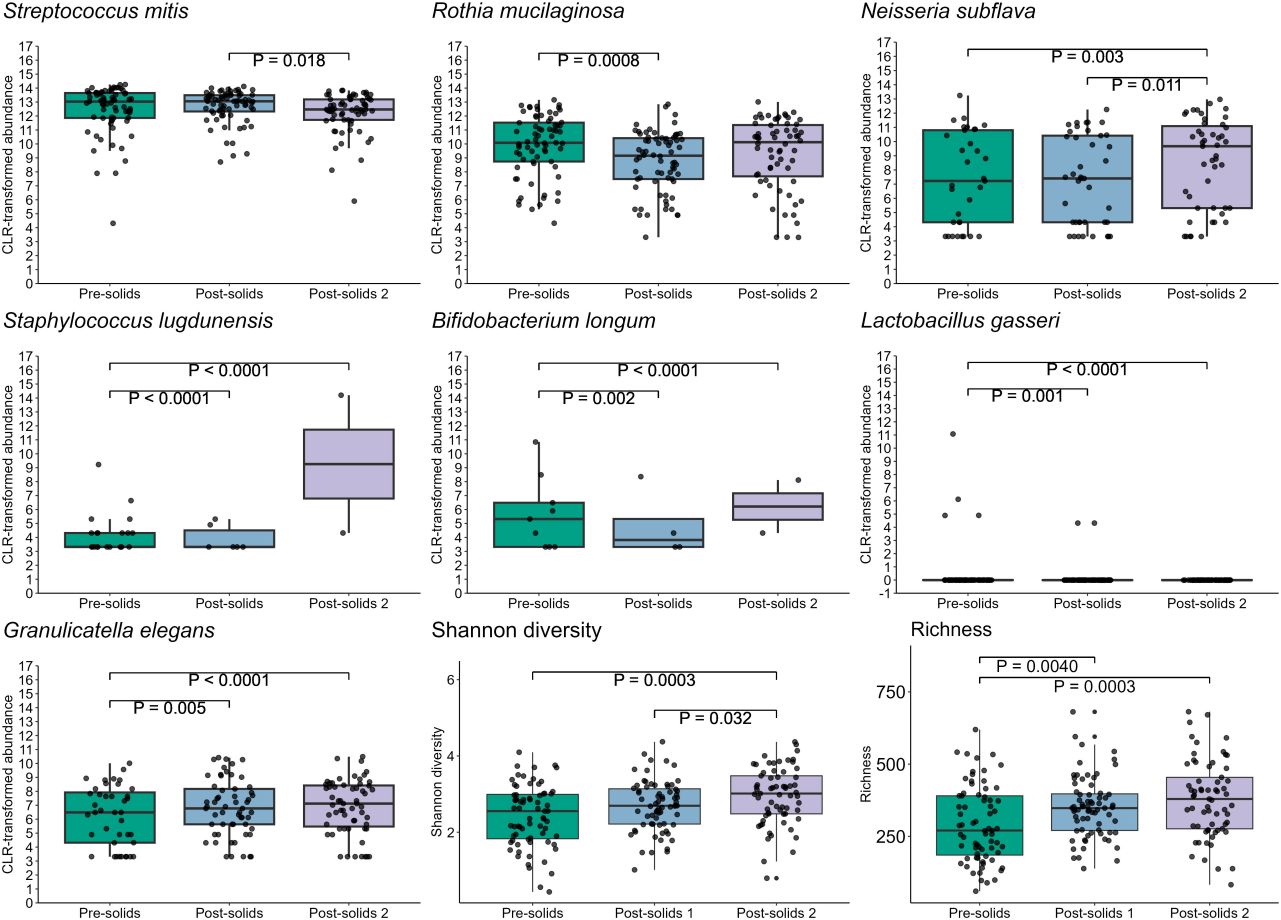
Introduction of solid foods is associated with multiple shifts in the infant oral microbiome.

Introduction of solid foods significantly impacted the infant oral microbiome (PERMANOVA, P = 0.001, R^2^ = 0.023), resulting in an increase in alpha bacterial diversity (richness: P = 0.0004, Shannon diversity: P = 0.0007) (**Figure 10**). In the first sample collected after the introduction of solid food, reductions in the relative abundances of *R. mucilaginosa* (P = 0.0008), *S. lugdunensis* (P < 0.001), *B. longum* (P = 0.002), and *L. gasseri* (P = 0.001), and an increase in the relative abundance of *Granulicatella elegans* (P = 0.005) were observed. Additionally, some changes did not become apparent until two sample collection time points after the first exposure to food (*N. subflava*, P = 0.003; *S. mitis*, P = 0.018) (**Figure 10**).

**Figure 11:**
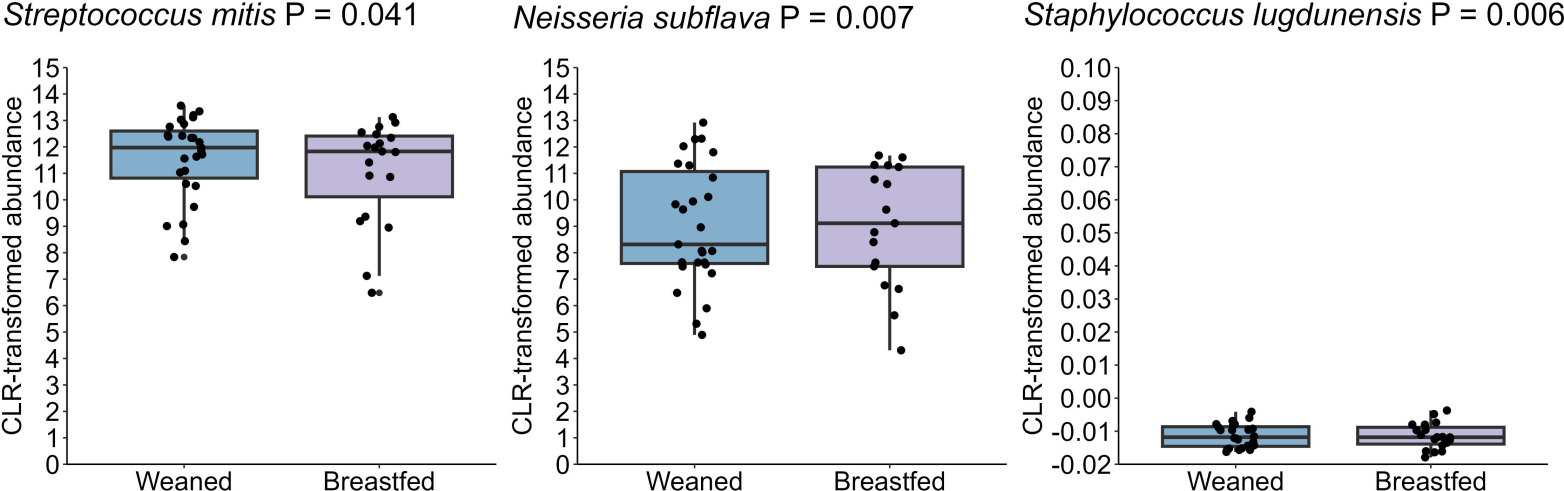
The oral microbiome at 24 months of age differs based on breastfeeding. Those who were weaned at this age had increased *S. mitis* and *S. lugdunensis* and reduced *N. subflava* (P = 0.041, P = 0.006, and P = 0.007, respectively).

In this cohort, all but two infants were still breastfed at 12 months of age, and 19 were still breastfed at 24 months of age. In order to assess the impact of complete cessation of breastfeeding on the infant oral microbiome, we compared 24-month samples from infants who were or were not receiving human milk. Infants who had ceased at 24 months (n= 27) exhibited an increase in *S. mitis* and *S. lugdunensis*, while showing a reduction in *N. subflava* (all P ≤ 0.05) compared to those who were still breastfeeding (n= 19) (**Figure 11**). However, neither alpha diversity nor beta diversity were different based on cessation of breastfeeding.

## 4 Discussion

Our longitudinal analysis of the infant oral microbiome in predominantly breastfed infants showed dynamic shifts in microbial diversity and abundance over the first two years of life. The oral microbiome was predominantly composed of taxa such as *S. mitis*, *G. haemolysans*, and *R. mucilaginosa*, aligning with previous studies (Cephas et al., 2011; Dzidic et al., 2018; Kennedy et al., 2019; Sulyanto et al., 2019; Li et al., 2023) and was impacted by various maternal, infant, and environmental factors.

The introduction of solid foods represents a pivotal milestone in infant development as this dietary shift not only provides new substrates for bacterial metabolism but also gradually reduces the intake of human milk, potentially altering the composition of the oral microbiome (Oba et al., 2020). This dietary change increases the availability of polysaccharides, favoring the growth of taxa that efficiently utilize these complex carbohydrates (Takahashi, 2015). The significant shift in nutrient sources that occurs with the introduction of solid foods is expected to impact the diversity and composition of the oral microbiome (Dzidic et al., 2018). Our study revealed significant shifts in the abundances of *S. mitis*, *R. mucilaginosa*, *N. subflava*, *S. lugdunensis*, *B. longum*, *L. gasseri*, and *G. elegans* after the introduction of food. Our results match those previously reported in two small studies (n=9 and n=12), both of which identified a decrease in *S. mitis* following the first intake of solids foods (Sulyanto et al., 2019; Oba et al., 2020). These studies also reported an increase in Shannon diversity and richness following the introduction of solid foods (Sulyanto et al., 2019), as well as an increase in *Gemella*, *Neisseria*, *Veillonella*, and *Fusobacterium* (Oba et al., 2020). Another study examining the oral microbiome of infants and their mothers found that incorporation of solid foods into the infant diet was associated with a compositional shifts in the oral microbiome, resulting in clustering patterns that more closely resemble those of their mothers rather than those of other infants (Cephas et al., 2011). These findings highlight the critical role of dietary factors in shaping the development of the oral microbial community. Given that early colonization shapes later waves of succession in microbial communities, more research attention should be given to the study of the effect of diet on the early oral microbiome.

The complete cessation of breastfeeding represents another dietary transition for the infant/child. In our study, the duration of breastfeeding was relatively long; however, infants who ceased breastfeeding by 24 months exhibited an increase in *S. mitis* and *S. lugdunensis*, while showing a reduction in *N. subflava* compared to those who were still breastfeeding. Dzidic et al (Dzidic et al., 2018). observed that infants breastfed for at least 12 months showed distinct oral microbiome profiles compared to those who ceased earlier, with persistent differences detected up to 7 years of age (Dzidic et al., 2018). These findings, although not directly comparable, highlight the role of human milk in shaping the infant oral microbiome beyond the first year of life. Interestingly, *S. mitis* abundance decreases following the introduction of solid foods, suggesting a preference for a breastfed-exclusive environment. However, by 24 months, *S. mitis* is slightly less abundant in breastfed infants compared to those who ceased infants. This pattern may reflect shifts in microbial community dynamics, competition with other taxa, or changes in oral environmental factors such as pH, salivary composition, and dietary substrates (Sedghi et al., 2021). While the underlying mechanisms remain unclear, these findings highlight the complex interactions between diet and the oral microbiome throughout early life and warrant further investigation.

Pacifiers are commonly employed to soothe infants and are often introduced early in life; however, emerging research suggests associations with alterations in oral microbial composition. Our study revealed a higher abundance of *S. salivarius* group 1 and *S. parasanguinis* in the oral cavities of infants who used pacifiers in the first week of life. This increase may be linked to the mechanical interaction between the pacifier, saliva, and the oral microbiome, which could create favorable conditions for biofilm formation (Brook and Gober, 1997; Comina et al., 2006). Notably, *Streptococcus* species preferentially form biofilms, as they account for 80% of the early colonizers during the initial adhesion phase, relying primarily on oral glycoproteins and salivary mucins (Radaic and Kapila, 2021). Additionally, the act of sucking on a pacifier can enhance saliva production and alter its flow and distribution, thereby creating an environment for bacteria that thrive in saliva-rich conditions (Comina et al., 2006). Two studies have identified a significant positive association between pacifier use and the abundance of lactobacilli and *Candida* spp. in the oral microbiome of breastfed infants (Ollila et al., 1997; Ersin et al., 2006). However, it is important to note that pacifier use was only recorded for the first week of life in our cohort. Whilst it is likely that use of the pacifier in these infants was continued, we do not have data to support this, thus our findings should be regarded as preliminary and should be verified in other cohorts.

Intrapartum antibiotic prophylaxis is administered for caesarean section delivery, to prevent Group B Streptococcus (GBS) infection, and for prolonged rupture of membranes (PROM). Approximately 20% of women test positive for GBS in the third trimester of pregnancy; however, not all of them consent to intrapartum antibiotic prophylaxis (Le Doare et al., 2017). Despite its widespread use, there has been limited comprehensive investigation into the immediate and long-term effects of perinatal antibiotic administration on the infant oral microbiome (Kuperman and Koren, 2016). Our findings revealed that infants born to antibiotic-exposed mothers, harbored oral microbiomes with reduced abundance of *S. lugdunensis* compared to infants born to non-exposed mothers. Interestingly this differs from two other studies that analyzed the microbiome at the family level showing a higher abundance of *Proteobacteria* in exposed infants, while unexposed displayed a dominance of *Streptococcaceae*, *Gemellaceae*, and *Lactobacillales* (Gomez-Arango et al., 2017). In another study, unexposed infants having elevated levels of Firmicutes and decreased levels of *Actinobacteria*, *Bacteroidetes*, and *Proteobacteria* compared to the exposed group (Li et al., 2019). Further large studies are required to unravel the effects of intrapartum antibiotic use in breastfed infants, with a focus on the class of antibiotic used.

Delivery mode is considered a major covariate in the establishment of oral bacteria in infants and has been explored in various studies (Li et al., 2005, 2018; Holgerson et al., 2011; Nelun Barfod et al., 2011; Thakur et al., 2012; Dominguez-Bello et al., 2016; Hurley et al., 2019); however, the differential effects of planned and intrapartum caesareans remain under explored. We observed lower Shannon diversity and richness in the oral cavities of infants delivered via planned, but not intrapartum, caesarean section. Further, whilst previous studies have reported compositional differences in the oral microbiome between vaginally born infants and those delivered by caesarean section (Li et al., 2005, 2018; Holgerson et al., 2011; Nelun Barfod et al., 2011; Thakur et al., 2012; Dominguez-Bello et al., 2016; Hurley et al., 2019), we found no such differences when conducting full-length sequencing of the 16S rRNA gene.

Interestingly, our findings suggest that the type of caesarean delivery is important, with differences seen in infants delivered via planned, but not intrapartum, caesarean section. Although this distinction has not been investigated in the context of the infant oral microbiome previously, our findings do match up with observations in the infant gut microbiome, where infants born via intrapartum caesarean more closely resemble those born vaginally (Penders et al., 2006; Stokholm et al., 2016; Liu et al., 2023; Weng et al., 2023; Yu et al., 2024). These findings underscore the necessity of analyzing delivery mode as a three-class variable for infant microbiome studies.

Maternal BMI has been identified as a potential determinant of the infant oral microbiome. In our study, we found significantly higher relative abundances of *N. subflava* and *Prevotella* sp. in infants born to mother with underweight/normal-weight compared to those born to mothers with overweight or obesity. While the reason for this association is unknown, previous studies have found that the human milk microbiome differs based on maternal BMI (Lundgren et al., 2019; LeMay-Nedjelski et al., 2020; Cortés-Macías et al., 2021). Additionally, the infant oral microbiome is likely influenced by bioactive components, such as immune proteins, in milk, which may also be impacted by maternal BMI (Cortés-Macías et al., 2021). More in depth studies of multiple milk components may aid in interpreting the impact of maternal body composition on infant microbiome colonization.

Our investigation also explored, for the first time, the potential influence of household pets on the infant oral microbiome. Dogs and cats, known to harbor diverse microbial communities, have been associated with changes in the human gut microbiome (Azad et al., 2013; Tun et al., 2017). Our results revealed a lower abundance of *Prevotella* spp. in the oral microbiome of infants living with pets compared to those in pet-free homes. Although this finding does not fully align with previous studies on the infant gut microbiome, where exposure to furry pets has been linked to increased microbial diversity (Azad et al., 2013; Tun et al., 2017; Panzer et al., 2023), it suggests that similar mechanisms may influence the oral microbiome. Pets can harbor and transmit various microbes. Microbial transmission to infants may occur through direct contact with pets or via intermediate reservoirs in the home, such as house dust or the maternal microbiome (Azad et al., 2013; Konya et al., 2014) highlighting the need for further investigation.

Birth season has also been recognized as a potential determinant in shaping the development of the oral microbiome during infancy however no studies have investigated this. We found that infants born in winter or autumn exhibited a lower relative abundance of *S. parasanguinis.* In contrast, a study of the infant nasopharyngeal microbiome found that season of birth was linked to the diversity of bacteria in infants’ nasopharynx at one month of age, with greater richness and altered taxonomic composition observed among those born in summer (Schoos et al., 2020). These findings suggest that birth season may influence microbial colonization in different body sites, indicating a broader seasonal impact on microbial communities. Further research is needed to explore these seasonal variations in the oral microbiome and their potential implications for infant health, particularly in light of association between birth season and infant allergic disease (Kuwabara et al., 2020; Tsuchida et al., 2023).

The key strengths of this study are its longitudinal nature, allowing identification of temporal patterns over 24 months, and the relatively large sample size compared to previous similar studies. In addition, the use of full-length 16S rRNA gene sequencing, offers advantages over short-read sequencing techniques, including improved taxonomic resolution and accuracy in microbial identification.

Several limitations, however, must be acknowledged. Firstly, the precise timing of solid food introduction was not recorded, nor were the types and quantities of food to which infants were exposed to. However, our study partly addressed this limitation by analyzing data at two subsequent time points after the introduction of solid foods. Secondly, the timing of tooth eruption was not documented. Tooth eruption and the introduction of solid foods often occur around the same time and may influence the composition and diversity of the oral microbiome (Sulyanto et al., 2019). Additionally, daycare attendance data were collected only at 12 months as a binary variable (yes/no), with substantial missing data and no information on attendance frequency or location. Due to these limitations, it was not included in our modelling; however, it should be considered in future studies.

In summary, this study represents one of the most comprehensive examinations of the infant oral microbiome with respect to breastfeeding conducted to date. Our findings illustrate the dynamic nature of the oral microbiome over the first two years of life, with significant associations identified with multiple factors, including pacifier use, intrapartum antibiotic prophylaxis, maternal allergy, pre-pregnancy maternal BMI, delivery mode, maternal age, presence of pets at home, siblings, and birth season. We demonstrated that the introduction of solid foods is a significant milestone in the development of the oral microbiome along with cessation of breastfeeding. The impact of these changes on infant health and development requires further investigation.

## Data Availability

The datasets presented in this study are available at the NCBI Short Read Archive, accession number PRJNA1186108.
