# Supplementary material for: Development of the breastfed infant oral microbiome over the first two years of life in the BLOSOM Cohort
Source: Front Cell Infect Microbiol. 2025 Apr 15;15:1534750. doi: 10.3389/fcimb.2025.1534750 (PMC12037575; doi:10.3389/fcimb.2025.1534750)
Supplement: Supplementary file 1 [file Table1.docx]

## Supplementary tables

**Supplementary Table S1**: Genera detected in negative extraction controls (EC, n = 8) and negative PCR controls (NTC, n = 8). Data are read numbers.

| Genus | **EC1** | **EC2** | **EC3** | **EC4** | **EC5** | **EC6** | **EC7** | **EC8** | **NTC1** | **NTC2** | **NTC3** | **NTC4** | **NTC5** | **NTC6** | **NTC7** | **NTC8** |
| --- | --- | --- | --- | --- | --- | --- | --- | --- | --- | --- | --- | --- | --- | --- | --- | --- |
| *Actinomyces* | 2 | 2 | 0 | 0 | 3 | 1 | 1 | 0 | 1 | 1 | 0 | 0 | 1 | 1 | 0 | 0 |
| *Atopobium* | 2 | 1 | 0 | 1 | 0 | 0 | 0 | 0 | 0 | 1 | 2 | 0 | 0 | 0 | 0 | 0 |
| *Bergeyella* | 7 | 1 | 5 | 0 | 1 | 2 | 1 | 1 | 0 | 1 | 8 | 1 | 2 | 1 | 4 | 0 |
| *Gemella* | 37 | 0 | 1 | 0 | 0 | 0 | 1 | 1 | 1 | 0 | 1 | 2 | 2 | 0 | 3 | 1 |
| *Granulicatella* | 1 | 3 | 0 | 0 | 2 | 2 | 1 | 5 | 1 | 1 | 1 | 1 | 2 | 2 | 1 | 2 |
| *Haemophilus* | 3 | 0 | 0 | 0 | 0 | 0 | 0 | 0 | 0 | 0 | 0 | 0 | 0 | 0 | 0 | 0 |
| *Lactobacillus* | 1 | 1 | 1 | 0 | 1 | 1 | 0 | 0 | 0 | 1 | 3 | 0 | 1 | 1 | 0 | 1 |
| *Neisseria* | 8 | 2 | 5 | 2 | 5 | 4 | 1 | 2 | 0 | 4 | 2 | 1 | 9 | 2 | 2 | 3 |
| *Porphyromonas* | 9 | 0 | 0 | 0 | 0 | 0 | 0 | 0 | 0 | 1 | 1 | 0 | 2 | 1 | 0 | 0 |
| *Rothia* | 70 | 3 | 2 | 1 | 1 | 4 | 1 | 1 | 3 | 1 | 2 | 0 | 3 | 1 | 2 | 3 |
| Unclassified *Saccharimonadales* | 4 | 4 | 8 | 1 | 11 | 7 | 5 | 26 | 3 | 10 | 10 | 6 | 7 | 7 | 11 | 1 |
| *Sebaldella* | 1 | 0 | 0 | 0 | 0 | 1 | 0 | 0 | 0 | 0 | 0 | 0 | 0 | 0 | 0 | 0 |

| Genus | **EC1** | **EC2** | **EC3** | **EC4** | **EC5** | **EC6** | **EC7** | **EC8** | **NTC1** | **NTC2** | **NTC3** | **NTC4** | **NTC5** | **NTC6** | **NTC7** | **NTC8** |  |
| --- | --- | --- | --- | --- | --- | --- | --- | --- | --- | --- | --- | --- | --- | --- | --- | --- | --- |
| *Staphylococcus* | 3 | 0 | 3 | 0 | 6 | 5 | 1 | 0 | 2 | 4 | 1 | 2 | 3 | 2 | 5 | 0 |  |
| *Streptococcus* | 719 | 13 | 18 | 7 | 27 | 16 | 11 | 60 | 6 | 11 | 21 | 16 | 21 | 15 | 13 | 4 |  |
| Unclassified *Actinobacteria* | 2 | 2 | 0 | 0 | 0 | 3 | 1 | 0 | 0 | 0 | 3 | 1 | 1 | 1 | 1 | 0 |  |
| Unclassified *Bacilli* | 3 | 1 | 1 | 0 | 2 | 3 | 2 | 0 | 0 | 0 | 2 | 0 | 1 | 2 | 0 | 1 |  |
| Unclassified bacteria | 37 | 2 | 1 | 0 | 2 | 5 | 3 | 0 | 1 | 6 | 4 | 2 | 6 | 1 | 2 | 1 |  |
| Unclassified *Carnobacteriaceae* | 3 | 1 | 2 | 1 | 2 | 8 | 1 | 0 | 4 | 4 | 7 | 1 | 4 | 5 | 3 | 2 |  |
| Unclassified *Lactobacillales* | 7 | 4 | 2 | 1 | 12 | 18 | 6 | 0 | 0 | 1 | 3 | 4 | 9 | 8 | 11 | 3 |  |
| Unclassified *Leptotrichiaceae* | 1 | 1 | 0 | 0 | 3 | 0 | 0 | 3 | 1 | 0 | 2 | 0 | 2 | 0 | 1 | 0 |  |
| Unclassified *Micrococcaceae* | 6 | 0 | 0 | 0 | 0 | 0 | 0 | 0 | 0 | 0 | 2 | 0 | 0 | 0 | 0 | 0 |  |
| Unclassified *Micrococcales* | 25 | 5 | 6 | 2 | 2 | 9 | 2 | 4 | 3 | 4 | 8 | 6 | 8 | 4 | 4 | 2 |  |
| Unclassified *Neisseriaceae* | 1 | 0 | 4 | 2 | 3 | 4 | 61 | 9 | 0 | 0 | 0 | 0 | 0 | 1 | 1 | 2 |  |
| Unclassified *Porphyromonadaceae* | 1 | 3 | 1 | 0 | 4 | 1 | 3 | 1 | 1 | 2 | 0 | 1 | 7 | 2 | 4 | 0 |  |
| Unclassified *Streptococcaceae* | 33 | 1 | 4 | 0 | 2 | 0 | 0 | 0 | 0 | 1 | 1 | 0 | 0 | 0 | 2 | 0 |  |
| *Veillonella* | 549 | 8 | 9 | 2 | 6 | 7 | 6 | 18 | 3 | 8 | 4 | 11 | 8 | 8 | 5 | 2 |  |
| Others* | 1 | 7 | 6 | 9 | 51 | 57 | 95 | 33 | 6 | 13 | 4 | 9 | 41 | 47 | 27 | 15 |  |

*“Others” represents genera with ≤ 26 reads in negative control

**Supplementary Table S2**: Outputs from the univariate linear model examining associations between infant age and the oral microbiome (reference = 1 month). Significant associations are reported.

| Taxa | Infant age | Coefficient | Standard error | P-value | Adjusted P-value |
| --- | --- | --- | --- | --- | --- |
| *Streptococcus mitis* (OTU000001) | 3 months | 0.515 | 0.191 | 0.007 | **0.017** |
|  | 12 months | -0.510 | 0.203 | 0.012 | **0.028** |
|  | 24 months | -0.584 | 0.216 | 0.007 | **0.017** |
| *Gemella haemolysans* (OTU000002) | 1 week | 0.745 | 0.257 | 0.003 | **0.010** |
|  | 5 months | -0.849 | 0.255 | 0.0009 | **0.002** |
|  | 6 months | -0.726 | 0.260 | 0.005 | **0.014** |
|  | 9 months | -0.735 | 0.268 | 0.006 | **0.015** |
|  | 12 months | -0.700 | 0.274 | 0.011 | **0.025** |
| *Rothia mucilaginosa* (OTU000003) | 2 months | 1.044 | 0.263 | **< 0.001** | **0.0002** |
|  | 3 months | 1.751 | 0.263 | **< 0.001** | **< 0.001** |
|  | 4 months | 1.579 | 0.266 | **< 0.001** | **< 0.001** |
|  | 5 months | 1.602 | 0.259 | **< 0.001** | **< 0.001** |
|  | 6 months | 0.839 | 0.264 | 0.001 | **0.004** |
|  | 9 months | 0.815 | 0.273 | 0.002 | **0.007** |
|  | 12 months | 1.240 | 0.279 | **< 0.001** | **< 0.001** |
| *Streptococcus salivarius* group1 (OTU000004) | 3 months | -1.536 | 0.303 | **< 0.001** | **< 0.001** |
|  | 4 months | -1.496 | 0.306 | **< 0.001** | **< 0.001** |
|  | 5 months | -1.583 | 0.298 | **< 0.001** | **< 0.001** |
|  | 6 months | -1.483 | 0.304 | **< 0.001** | **< 0.001** |
|  | 9 months | -1.312 | 0.314 | **< 0.001** | **0.0001** |
| *Veillonella* sp. (OTU000005) | 1 week | -1.072 | 0.277 | 0.0001 | **0.0004** |
|  | 12 months | -1.356 | 0.296 | **< 0.001** | **< 0.001** |
| *Neisseria subflava* (OTU000006) | 6 months | 0.602 | 0.246 | 0.014 | **0.031** |
|  | 9 months | 2.155 | 0.254 | **< 0.001** | **< 0.001** |
|  | 12 months | 3.067 | 0.260 | **< 0.001** | **< 0.001** |
|  | 24 months | 2.685 | 0.277 | **< 0.001** | **< 0.001** |
| *Streptococcus oralis* (OTU000007) | 3 months | 0.905 | 0.254 | 0.0004 | **0.001** |
|  | 4 months | 1.549 | 0.257 | **< 0.001** | **< 0.001** |
|  | 5 months | 1.651 | 0.251 | **< 0.001** | **< 0.001** |
|  | 6 months | 1.677 | 0.256 | **< 0.001** | **< 0.001** |
|  | 9 months | 1.204 | 0.264 | **< 0.001** | **< 0.001** |
|  | 12 months | 0.760 | 0.270 | 0.005 | **0.013** |
|  | 24 months | -0.686 | 0.288 | 0.017 | **0.037** |
| *Veillonella nakazawae* (OTU000009) | 1 week | -1.337 | 0.253 | **< 0.001** | **< 0.001** |
|  | 12 months | -0.861 | 0.270 | 0.001 | **0.004** |
|  | 9 months | -0.704 | 0.264 | 0.007 | **0.018** |
|  | 24 months | -0.716 | 0.288 | 0.013 | **0.029** |
| *Staphylococcus lugdunensis* (OTU000010) | 1 week | 1.359 | 0.230 | **< 0.001** | **< 0.001** |
|  | 2 months | -0.715 | 0.232 | 0.002 | **0.006** |
|  | 3 months | -1.366 | 0.233 | **< 0.001** | **< 0.001** |
|  | 4 months | -1.238 | 0.235 | **< 0.001** | **< 0.001** |
|  | 5 months | -1.682 | 0.229 | **< 0.001** | **< 0.001** |
|  | 6 months | -1.898 | 0.233 | **< 0.001** | **< 0.001** |
|  | 9 months | -1.890 | 0.240 | **< 0.001** | **< 0.001** |
|  | 12 months | -2.154 | 0.246 | **< 0.001** | **< 0.001** |
|  | 24 months | -1.924 | 0.262 | **< 0.001** | **< 0.001** |
| *Haemophilus parainfluenzae* (OTU000011) | 6 months | -0.632 | 0.250 | 0.011 | **0.027** |
|  | 24 months | 0.841 | 0.281 | 0.002 | **0.007** |
| *Flavobacteriaceae* sp. (OTU000012) | 9 months | 0.679 | 0.245 | 0.005 | **0.014** |
|  | 12 months | 1.101 | 0.250 | **< 0.001** | **< 0.001** |
| *Streptococcus parasanguinis* (OTU000013) | 12 months | 1.188 | 0.254 | **< 0.001** | **< 0.001** |
|  | 24 months | 0.834 | 0.270 | 0.002 | **0.006** |
| *Prevotella* sp. (OTU000014) | 5 months | 0.911 | 0.210 | **< 0.001** | **< 0.001** |
|  | 6 months | 1.552 | 0.214 | **< 0.001** | **< 0.001** |
|  | 9 months | 1.191 | 0.220 | **< 0.001** | **< 0.001** |
|  | 12 months | 0.784 | 0.225 | 0.0005 | **0.001** |
| *Prevotella* sp. (OTU000014) | 24 months | 0.904 | 0.240 | 0.0001 | **0.0006** |
| *Bifidobacterium longum* (OTU000015) | 4 months | -0.516 | 0.172 | 0.002 | **0.007** |
|  | 5 months | -0.814 | 0.167 | **< 0.001** | **< 0.001** |
|  | 6 months | -0.986 | 0.171 | **< 0.001** | **< 0.001** |
|  | 9 months | -1.222 | 0.176 | **< 0.001** | **< 0.001** |
|  | 12 months | -1.251 | 0.180 | **< 0.001** | **< 0.001** |
|  | 24 months | -1.019 | 0.192 | **< 0.001** | **< 0.001** |
| *Lactobacillus gasseri* (OTU000016) | 1 week | -0.441 | 0.158 | **< 0.001** | **0.014** |
|  | 4 months | -0.660 | 0.162 | **< 0.001** | **0.0001** |
|  | 5 months | -0.926 | 0.157 | **< 0.001** | **< 0.001** |
|  | 6 months | -1.014 | 0.161 | **< 0.001** | **< 0.001** |
|  | 9 months | -1.290 | 0.166 | **< 0.001** | **< 0.001** |
|  | 12 months | -1.269 | 0.170 | **< 0.001** | **< 0.001** |
|  | 24 months | -0.981 | 0.181 | **< 0.001** | **< 0.001** |
| *Granulicatella elegans* (OTU000018) | 5 months | 0.892 | 0.173 | **< 0.001** | **< 0.001** |
|  | 6 months | 1.616 | 0.177 | **< 0.001** | **< 0.001** |
|  | 9 months | 1.702 | 0.182 | **< 0.001** | **< 0.001** |
|  | 12 months | 1.206 | 0.186 | **< 0.001** | **< 0.001** |
|  | 24 months | 1.356 | 0.199 | **< 0.001** | **< 0.001** |
| *Porphyromonas* sp. (OTU000019) | 6 months | 0.474 | 0.190 | 0.012 | **0.028** |
|  | 9 months | 0.455 | 0.195 | 0.0205 | **0.043** |

**Supplementary Table S3:** P-values from non-parametric tests comparing alpha diversity between subsequent time points over the first two years of life.

| Test | Alpha diversity measures | Time points | P - value | Adjusted P-value |
| --- | --- | --- | --- | --- |
| Kruskal-Wallis test | Shannon | Time (all time points) | < **0.0001** |  |
|  | Richness | Time (all time points) | < **0.0001** |  |
| Wilcoxon test | Shannon diversity | 1 week vs. 1 month | **0.007** | 0.069 |
|  |  | 1 month vs. 2 months | 0.186 | 0.325 |
|  |  | 2 months vs. 3 months | 0.239 | 0.325 |
|  |  | 3 months vs. 4 months | 0.449 | 0.450 |
|  |  | 4 months vs. 5 months | 0.288 | 0.325 |
|  |  | 5 months vs. 6 months | 0.331 | 0.325 |
|  |  | 6 months vs. 9 months | **0.022** | 0.100 |
|  |  | 9 months vs. 12 months | **0.048** | 0.147 |
|  |  | 12 months vs. 24 months | 0.177 | 0.325 |
|  | Richness | 1 week vs. 1 month | 0.058 | 0.111 |
|  |  | 1 month vs. 2 months | **0.043** | 0.111 |
|  |  | 2 months vs. 3 months | 0.061 | 0.111 |
|  |  | 3 months vs. 4 months | 0.311 | 0.312 |
|  |  | 4 months vs. 5 months | 0.299 | 0.258 |
|  |  | 5 months vs. 6 months | **0.048** | 0.111 |
|  |  | 6 months vs. 9 months | 0.087 | 0.131 |
|  |  | 9 months vs. 12 months | 0.208 | 0.258 |
|  |  | 12 months vs. 24 months | **0.038** | 0.111 |

**Supplementary Table S4**: Outputs from multivariate linear mixed effect models for associations between maternal, infant, and environmental factors and the infant oral microbiome. Infant age was included as an interaction for each variable. Variables with significant P-values, and those which underlie interactions with significant P-values, are reported. IAP: Intrapartum antibiotic prophylaxis.

| Response variable | Explanatory variable | Estimate | Standard error | P-value |
| --- | --- | --- | --- | --- |
| Shannon | Planned caesarean | -0.475 | 0.214 | **0.026** |
|  | 4 months: Planned caesarean | 0.595 | 0.301 | **0.048** |
|  | 4 months | -0.232 | 0.215 | 0.281 |
|  | 5 months: Planned caesarean | 0.574 | 0.293 | **0.050** |
|  | 5 months | -0.167 | 0.202 | 0.410 |
|  | 9 months: Planned caesarean | 0.987 | 0.313 | **0.001** |
|  | 9 months | 0.331 | 0.206 | 0.108 |
|  | 24 months: Planned caesarean | 0.775 | 0.339 | **0.022** |
|  | 24 months | 0.979 | 0.228 | **< 0.001** |
|  | 24 months: Birth season (autumn/ winter) | -0.567 | 0.264 | **0.0325** |
|  | Birth season (autumn/ winter) | 0.176 | 0.176 | 0.318 |
|  | 1 week: Birth season (autumn/ winter) | 0.492 | 0.236 | **0.037** |
|  | 1 week | -0.544 | 0.210 | **0.009** |
| Richness | Planned caesarean | -84.944 | 38.892 | **0.029** |
|  | 9 months: Planned caesarean | 191.471 | 57.494 | **< 0.001** |
|  | 9 months | 65.843 | 38.177 | 0.085 |
|  | 24 months: Planned caesarean | 122.239 | 61.591 | **0.047** |
|  | 24 months | 201.417 | 42.741 | **< 0.001** |
|  | 24 months: Birth season (autumn/ winter) | -125.079 | 47.089 | **0.008** |
|  | Birth season (autumn/ winter) | 39.987 | 31.401 | 0.203 |
| *Gemella haemolysans* (OTU000002) | 5 months: Birth season (autumn/ winter) | 3.046 | 0.903 | **< 0.001** |
|  | 5 months | -2.630 | 0.699 | **< 0.001** |
|  | 3 months: Birth season (autumn/ winter) | 2.175 | 0.926 | **0.019** |
|  | 3 months | -2.073 | 0.732 | **0.004** |
|  | Birth season (autumn/ winter) | -1.057 | 0.676 | 0.118 |
| *Rothia mucilaginosa* (OTU000003) | Time: 3 months | 4.231 | 0.772 | **< 0.001** |
|  | Time: 12 months | 3.517 | 0.779 | **< 0.001** |
|  | Time: 2 months | 3.007 | 0.777 | **0.0001** |
|  | Time: 9 months | 2.671 | 0.765 | **0.0005** |
|  | 1 week: birth season (autumn/ winter) | -3.265 | 0.970 | **0.0008** |
|  | 1 week | 0.564 | 0.769 | 0.463 |

| Response variable | Explanatory variable | Estimate | Standard error | P-value |
| --- | --- | --- | --- | --- |
| *Rothia mucilaginosa* (OTU000003) | 5 months: Birth season (autumn/ winter) | -2.741 | 0.954 | **0.004** |
|  | 5 months | 5.025 | 0.738 | **< 0.001** |
|  | 5 months | 5.025 | 0.738 | **< 0.001** |
|  | 4 months | 4.650 | 0.793 | **< 0.001** |
|  | 6 months | 3.530 | 0.757 | **< 0.001** |
|  | 24 months: Birth season (autumn/ winter) | -2.281 | 1.093 | **0.037** |
|  | 24 months | 2.211 | 0.846 | **0.009** |
|  | 2 months: Birth season (autumn/ winter) | -1.979 | 0.979 | **0.043** |
| *Streptococcus salivarius* group1 (OTU000004) | 5 months: Pets | -3.662 | 1.405 | **0.009** |
|  | 5 months | -0.084 | 1.125 | 0.940 |
|  | Pets | 0.065 | 1.131 | 0.954 |
|  | First week pacifier | 1.3620 | 0.622 | **0.031** |
| *Veillonella* sp. (OTU000005) | 24 months: First week pacifier | 4.121 | 1.579 | **0.009** |
|  | 24 months | -0.991 | 0.778 | 0.202 |
|  | First week pacifier | -1.924 | 1.128 | 0.088 |
| *Neisseria subflava* (OTU000006) | 5 months: IAP | -3.052 | 1.060 | **0.004** |
|  | 5 months | 3.418 | 0.693 | **< 0.001** |
|  | 4 months: IAP | -3.094 | 1.080 | **0.004** |
|  | 4 months | 1.939 | 0.714 | **0.006** |
|  | IAP | 0.972 | 0.850 | 0.253 |
|  | Pre- pregnancy maternal BMI (High/Obese) | 1.411 | 0.573 | **0.016** |
| *Streptococcus oralis* (OTU000007) | 5 months: Birth season (autumn/ winter) | -2.379 | 1.036 | **0.022** |
|  | 5 months | 5.451 | 0.802 | **< 0.001** |
|  | 4 months: Birth season (autumn/ winter) | -2.227 | 1.081 | **0.039** |
|  | 4 months | 4.989 | 0.862 | **< 0.001** |
|  | Birth season (autumn/ winter) | 0.731 | 0.844 | 0.386 |
|  | Time: 3 months | 1.724 | 0.838 | **0.040** |

| Response variable | Explanatory variable | Estimate | Standard error | P-value |
| --- | --- | --- | --- | --- |
| *Staphylococcus lugdunensis* (OTU000010) | 1 week: IAP | -3.663 | 0.918 | **< 0.001** |
|  | 1 week | 3.886 | 0.610 | **< 0.001** |
|  | 4 months: IAP | 2.423 | 0.928 | **0.009** |
|  | 4 months | -3.012 | 0.615 | **< 0.001** |
|  | 2 months: IAP | 2.322 | 0.931 | **0.012** |
|  | 2 months | -2.425 | 0.615 | **< 0.001** |
| *Staphylococcus lugdunensis* (OTU000010) | IAP | -1.523 | 0.670 | **0.023** |
| *Haemophilus parainfluenzae* (OTU000011) | 9 months: Birth season (autumn/ winter) | -2.872 | 1.171 | **0.014** |
|  | 9 months | 0.758 | 1.402 | 0.588 |
|  | Birth season (autumn/ winter) | -0.047 | 0.861 | 0.955 |
| *Streptococcus parasanguinis* (OTU000013) | First week pacifier | 1.571 | 0.535 | **0.004** |
|  | Birth season (autumn/ winter) | -1.205 | 0.439 | **0.007** |
| *Prevotella* sp. (OTU000014) | Maternal pre-pregnancy BMI (High/ Obese) | 1.222 | 0.396 | **0.002** |
|  | Pets | 0.903 | 0.375 | **0.018** |
| *Bifidobacterium longum* (OTU000015) | 5 months: Birth season (autumn/ winter) | -2.072 | 0.721 | **0.004** |
|  | 5 months | 0.223 | 0.558 | 0.688 |
|  | 4 months: Birth season (autumn/ winter) | -1.894 | 0.751 | **0.011** |
|  | 4 months | 0.641 | 0.599 | 0.285 |

## Supplementary figures


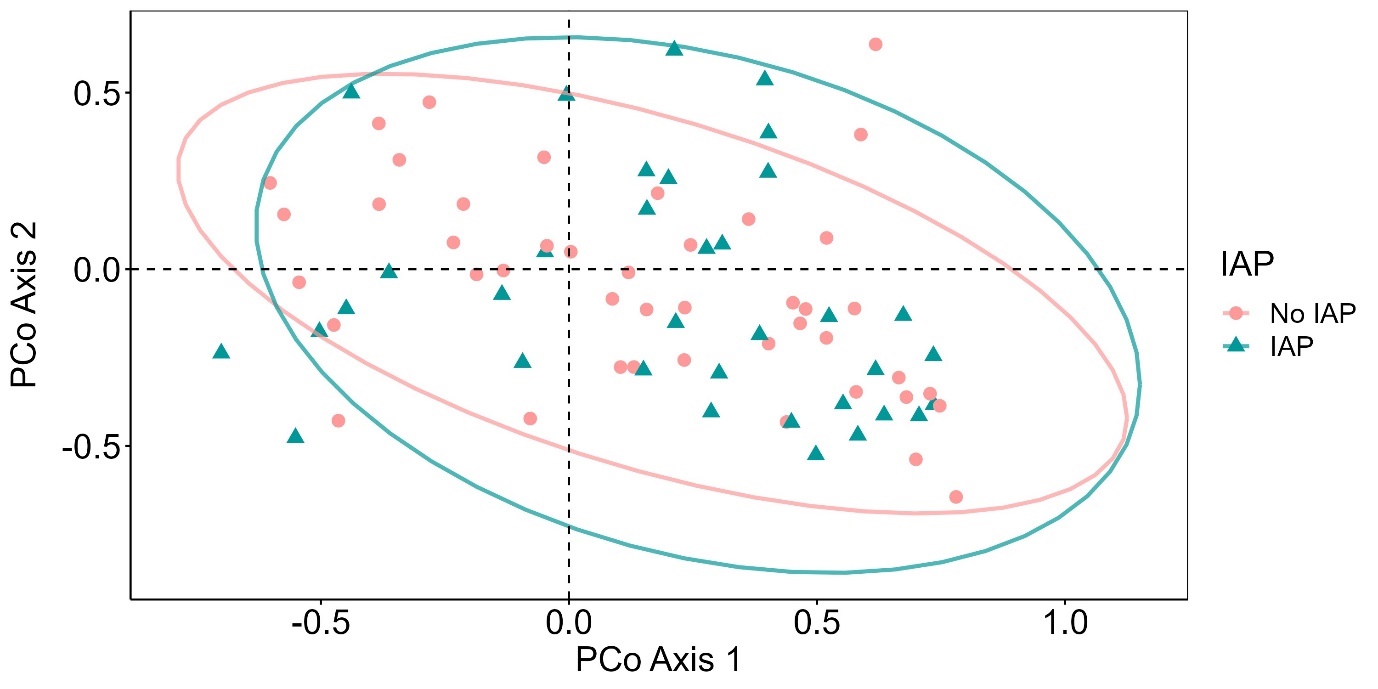


**Supplementary Figure S1.** PCoA plot of the beta diversity of the infant oral microbiome by maternal exposure to intrapartum antibiotic prophylaxis. Points represent infants: orange circles indicate antibiotic exposure, and blue triangles represent no exposure. Ellipses illustrate clustering within each group.


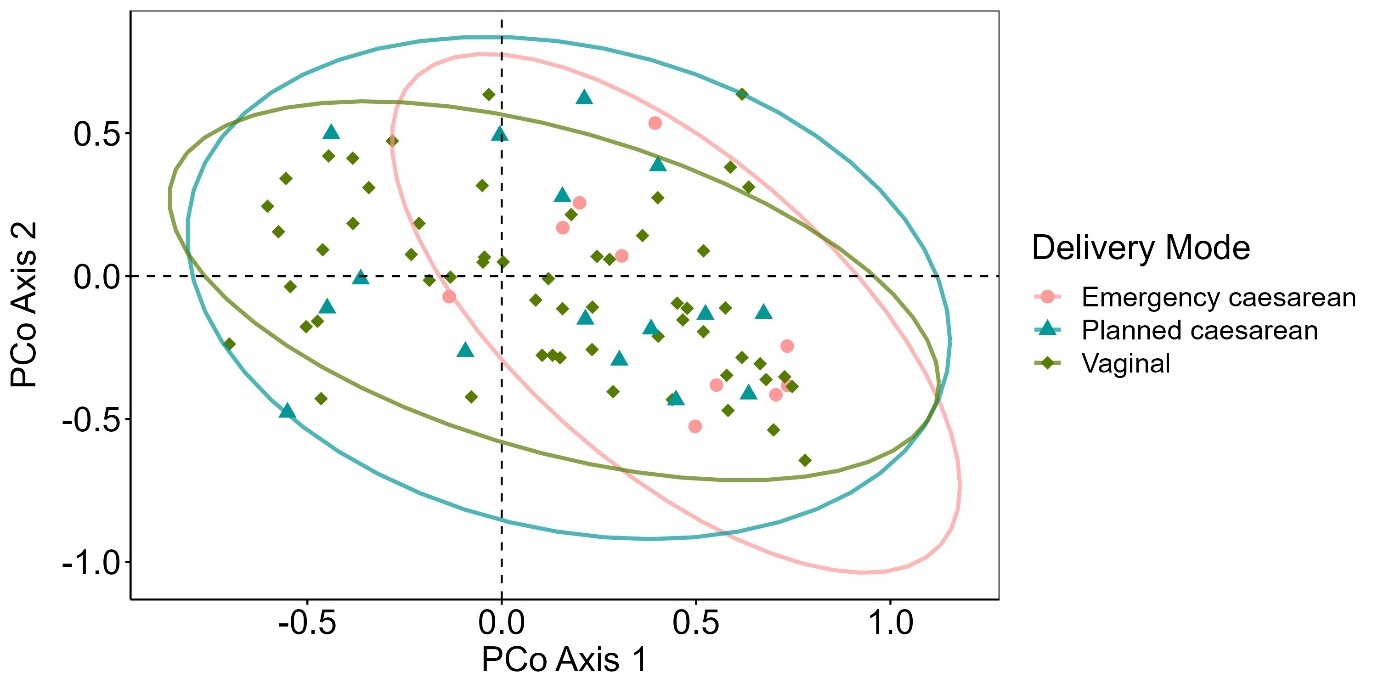


**Supplementary Figure S2**. **PCoA plot of the beta diversity of the infant oral microbiome by delivery mode.** Points represent infants: orange circles indicate those delivered by emergency caesarean, blue triangles represent those delivered by planned caesarean, and green circles indicate those delivered vaginally. Ellipses illustrate the clustering within each group.

**A**

A


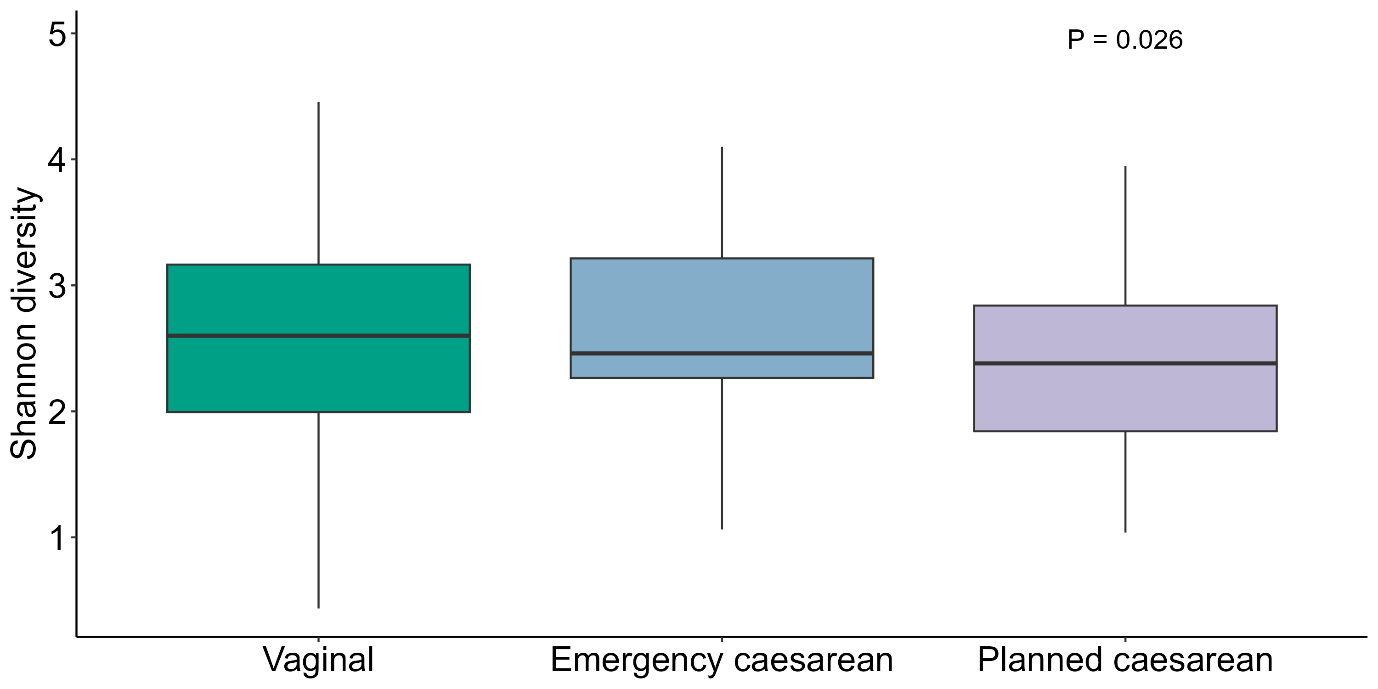


B


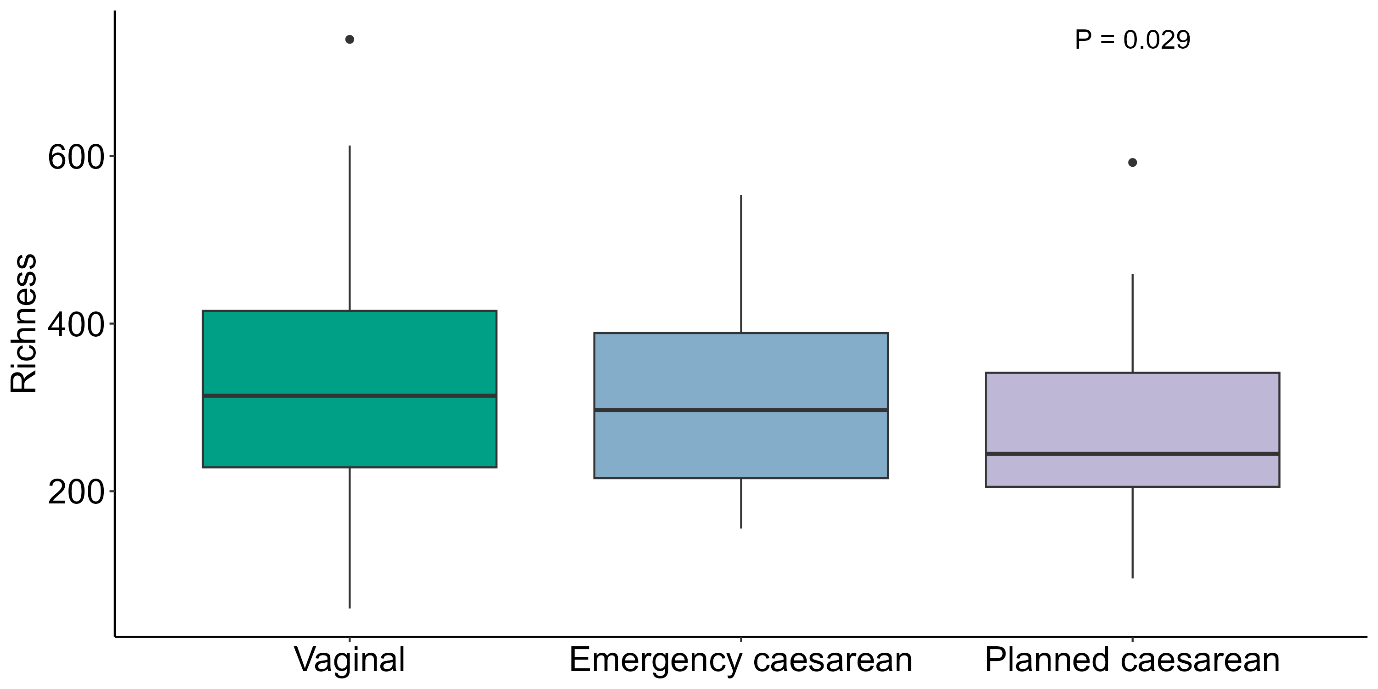


**Supplementary Figure S3**. Infants delivered by planned caesarean have significantly lower oral Shannon diversity (A) and richness (B).


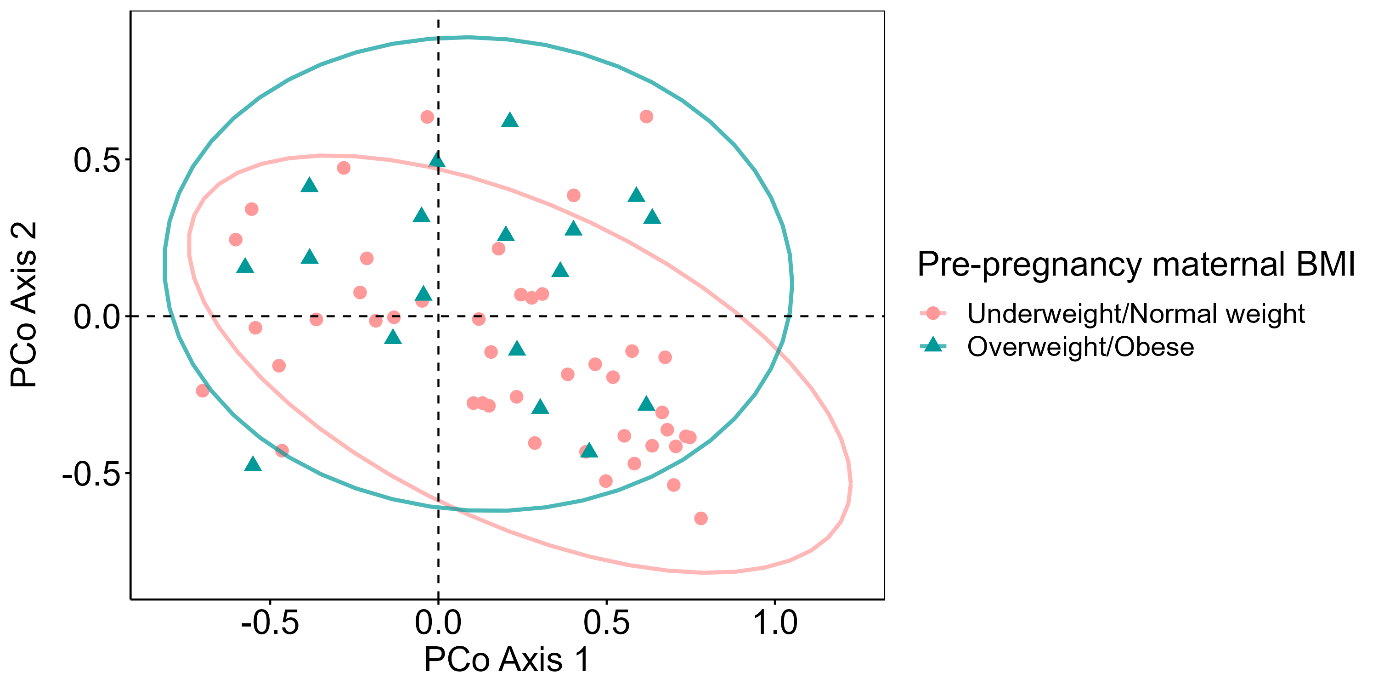


**Supplementary Figure S4.** PCoA plot of the beta diversity of the infant oral microbiome by pre-pregnancy maternal BMI. Points represent infants: orange circles indicate infants born to mothers with a normal-weight or underweight, while blue triangles represent infants born to mothers with overweight or obese. Ellipses illustrate the clustering within each group.


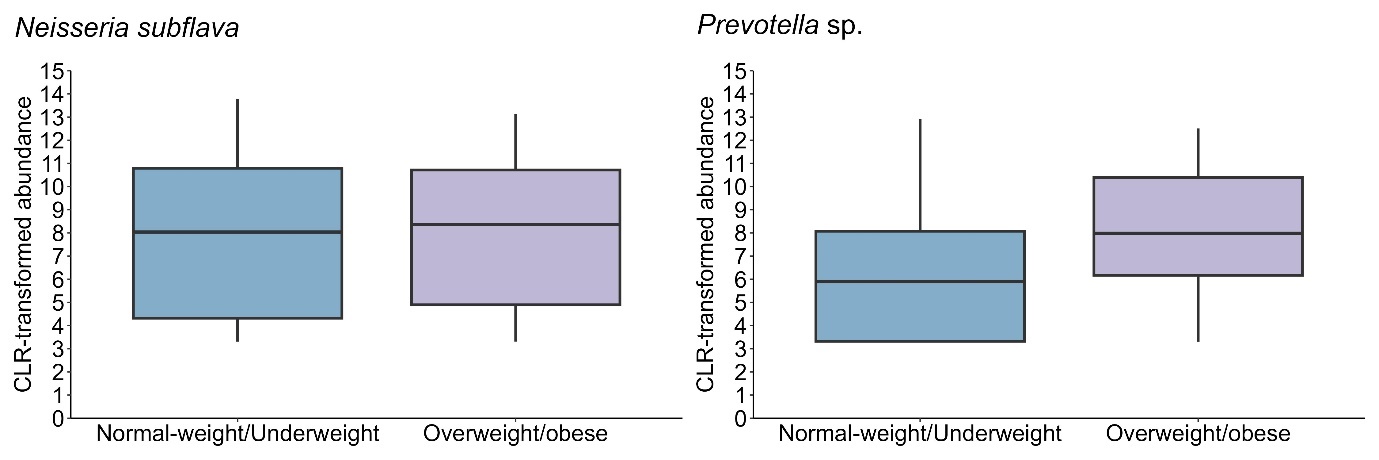


**Supplementary Figure S5**. A higher levels of *Neisseria subflava* and *Prevotella* sp. in the oral cavity of infants who born to mothers with higher weight and obesity (n = 18) compared to those born to underweight and normal-weight mothers (n = 44) (P = 0.016, P = 0.002, respectively). Data shown for the 1-month time point, with 1 month as the reference for age comparisons.


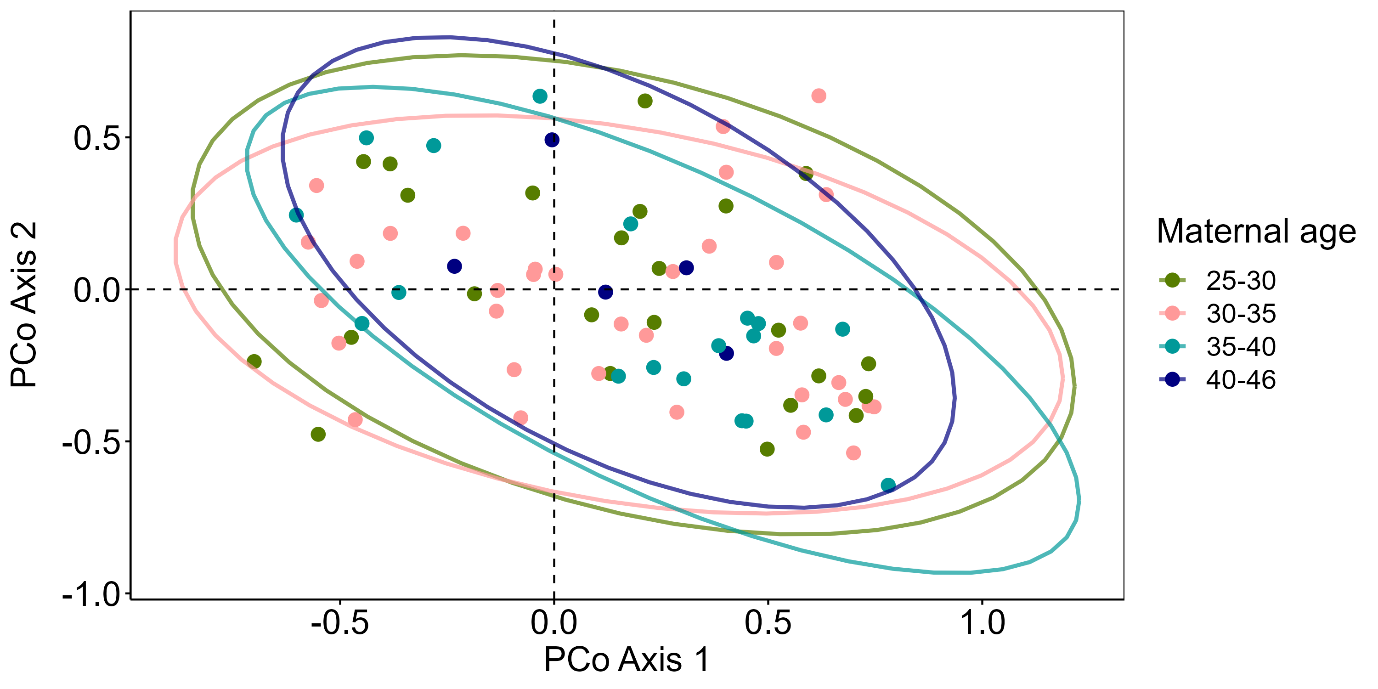


**Supplementary Figure S6.** PCoA plot of the beta diversity of the infant oral microbiome based on maternal age. Each point represents an infant, with the colour gradient indicating maternal age (from 25 to 46 years).


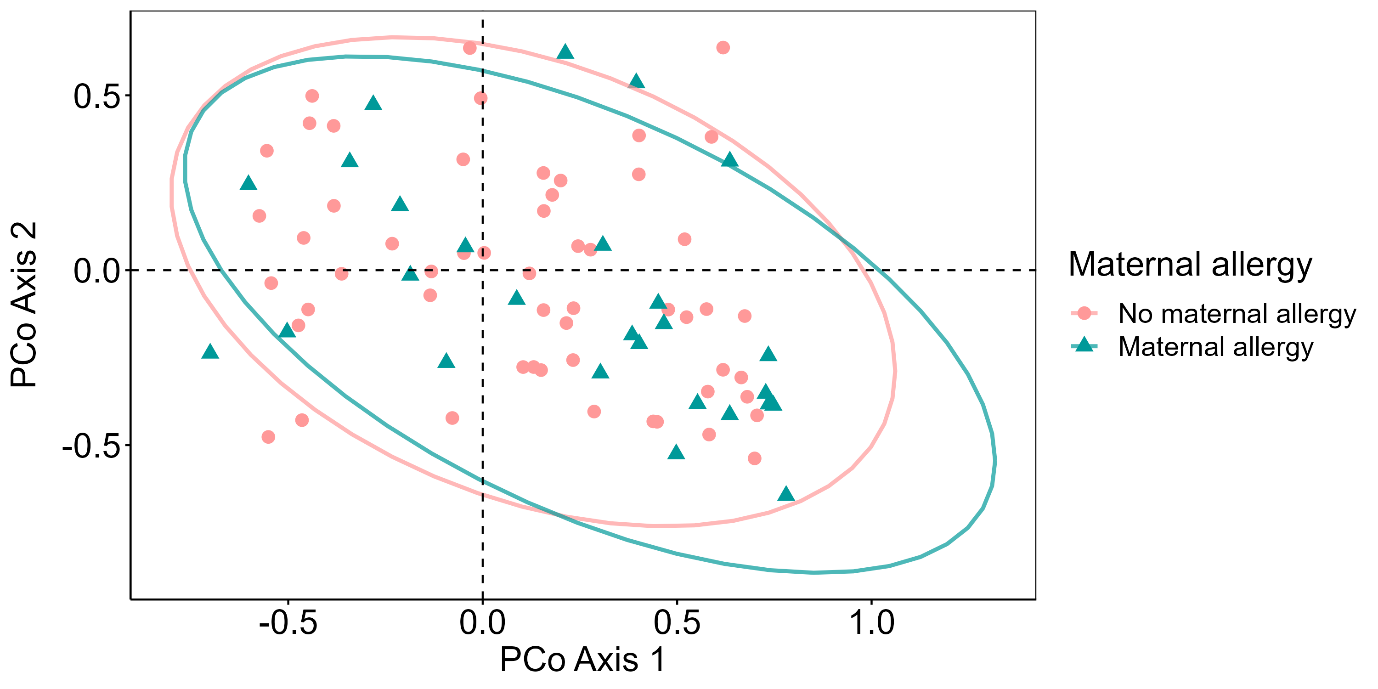


**Supplementary Figure S7.** PCoA plot of the beta diversity of the infant oral microbiome by maternal allergy status. Points represent infants: orange circles indicate infants born to allergic mother, while blue triangles represent those born to non-allergic mothers. Ellipses illustrate the clustering within each group.


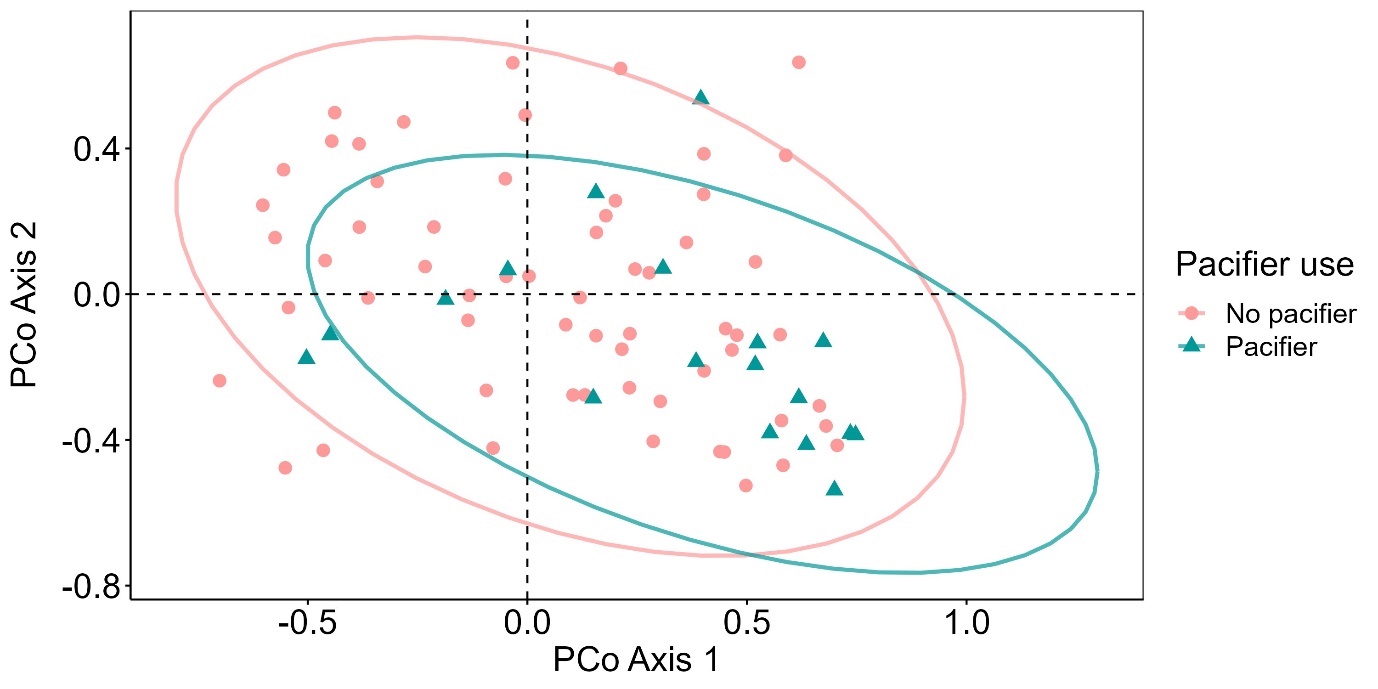


**Supplementary Figure S8.** PCoA plot of the beta diversity of the infant oral microbiome by pacifier use. Points represent infants: orange circles indicate infants who use pacifiers, while blue triangles represent those who do not use pacifiers. Ellipses illustrate the clustering within each group.


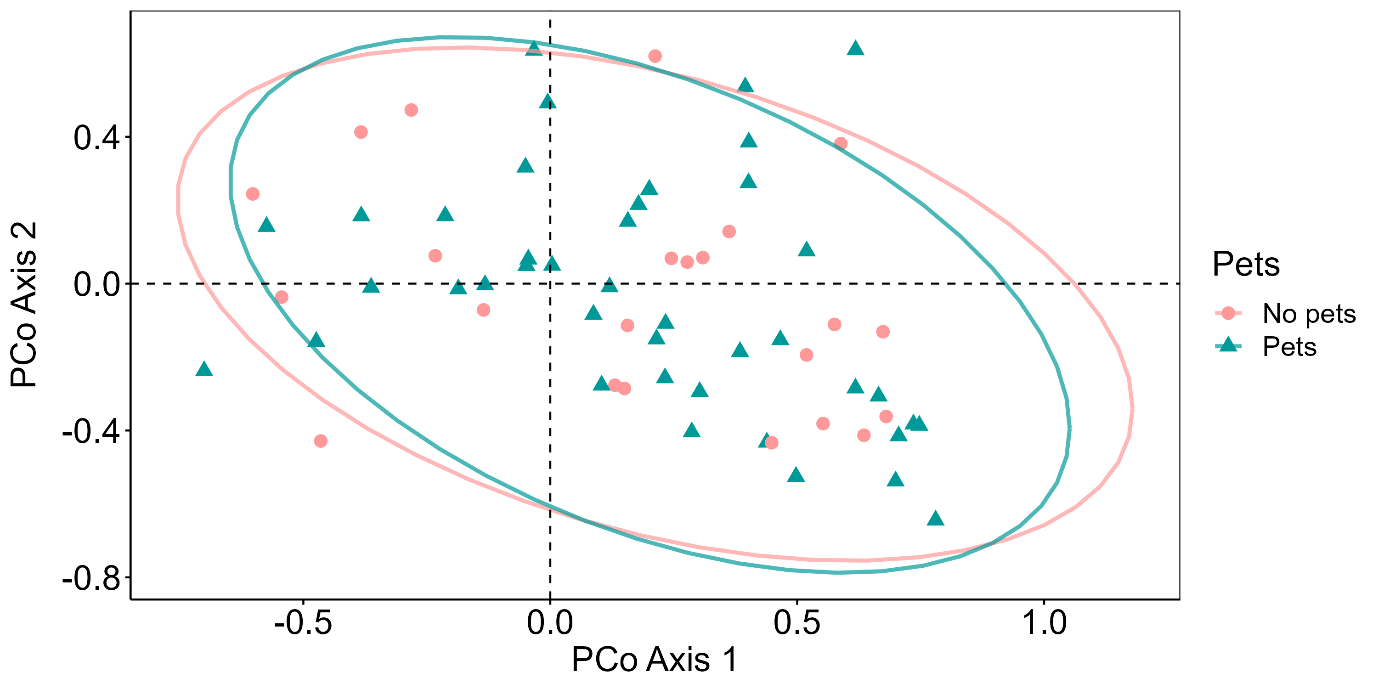


**Supplementary Figure S9.** PCoA plot of the beta diversity of the infant oral microbiome by presence of pets in the household. Points represent infants: orange circles indicate infants living in pet-free households, while blue triangles represent those living with pets. Ellipses illustrate the clustering within each group.


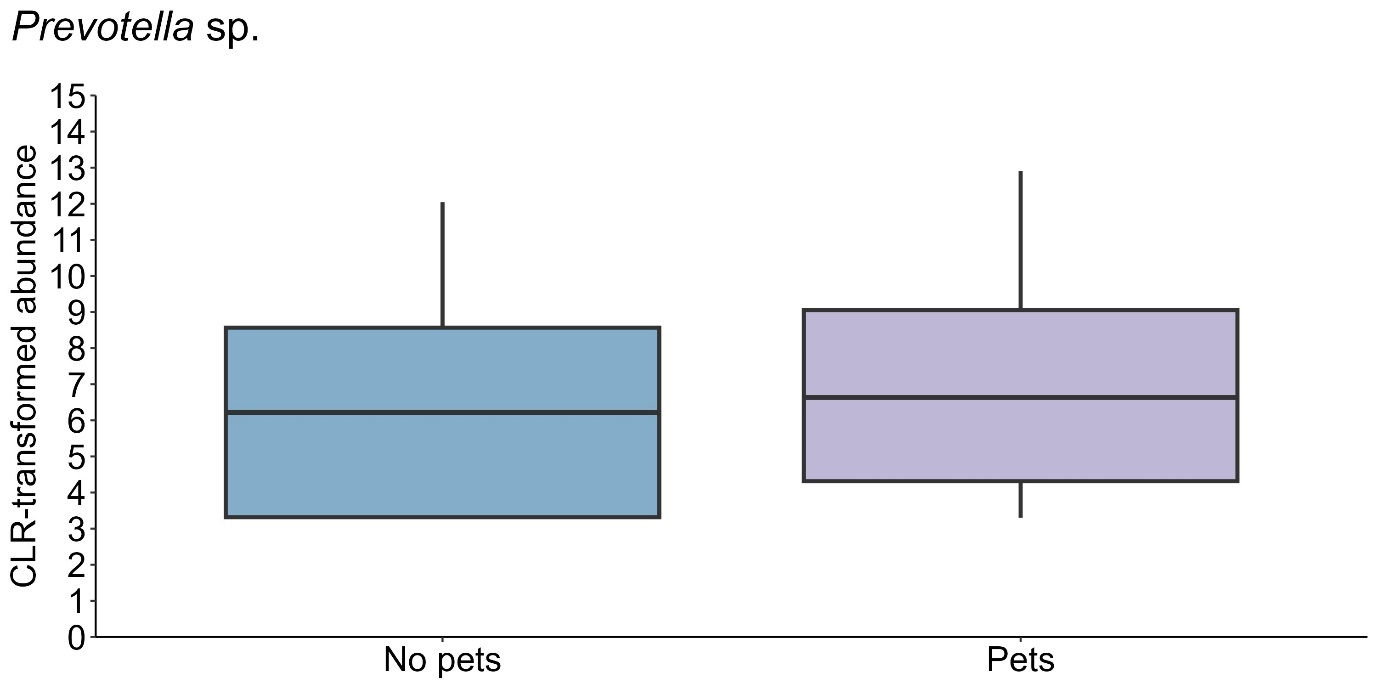


**Supplementary Figure S10.** A higher level of *Prevotella* sp. in the oral cavity of infants who raise in houses with furry pets (n = 48) versus those without pets (n = 16) (P = 0.018). Data shown for the 1-month time point, with 1 month as the reference for age comparisons.


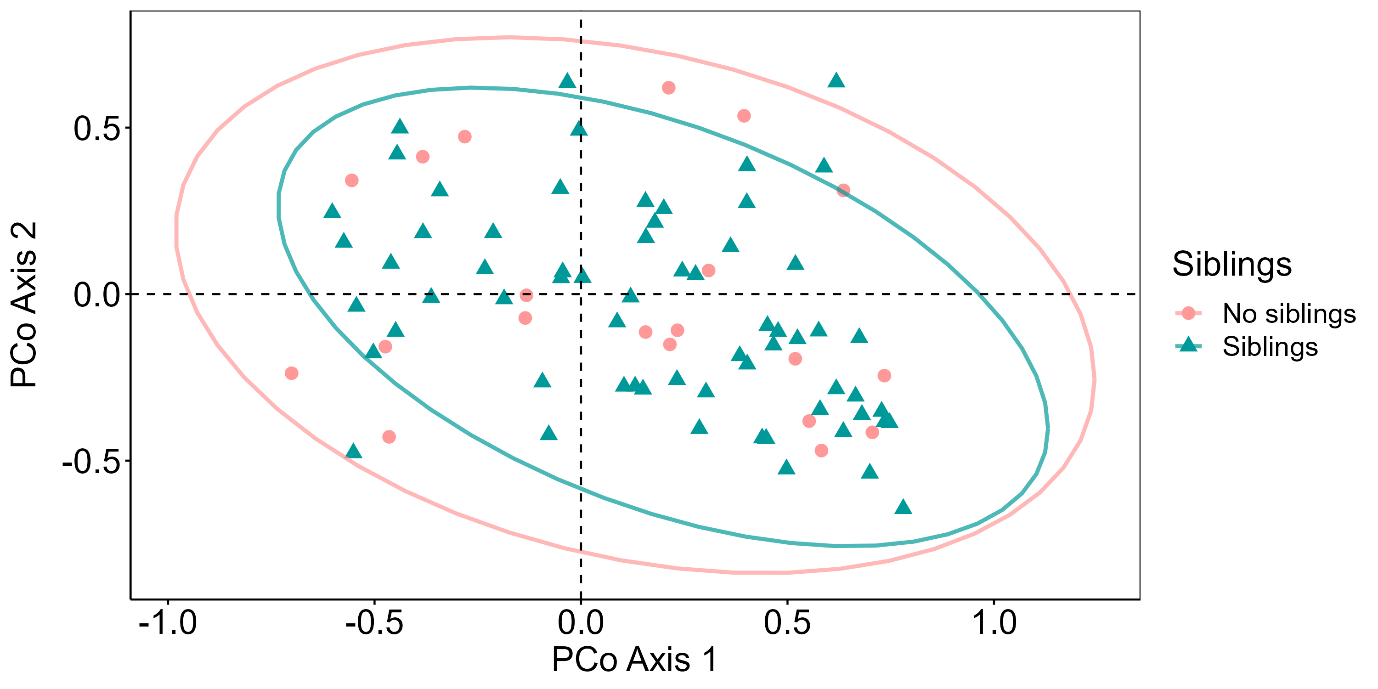


**Supplementary Figure S11.** PCoA plot of the beta diversity of the infant oral microbiome by sibling presence. Points represent infants: orange circles indicate infants with no siblings, while blue triangles represent those with siblings. Ellipses illustrate the clustering within each group.


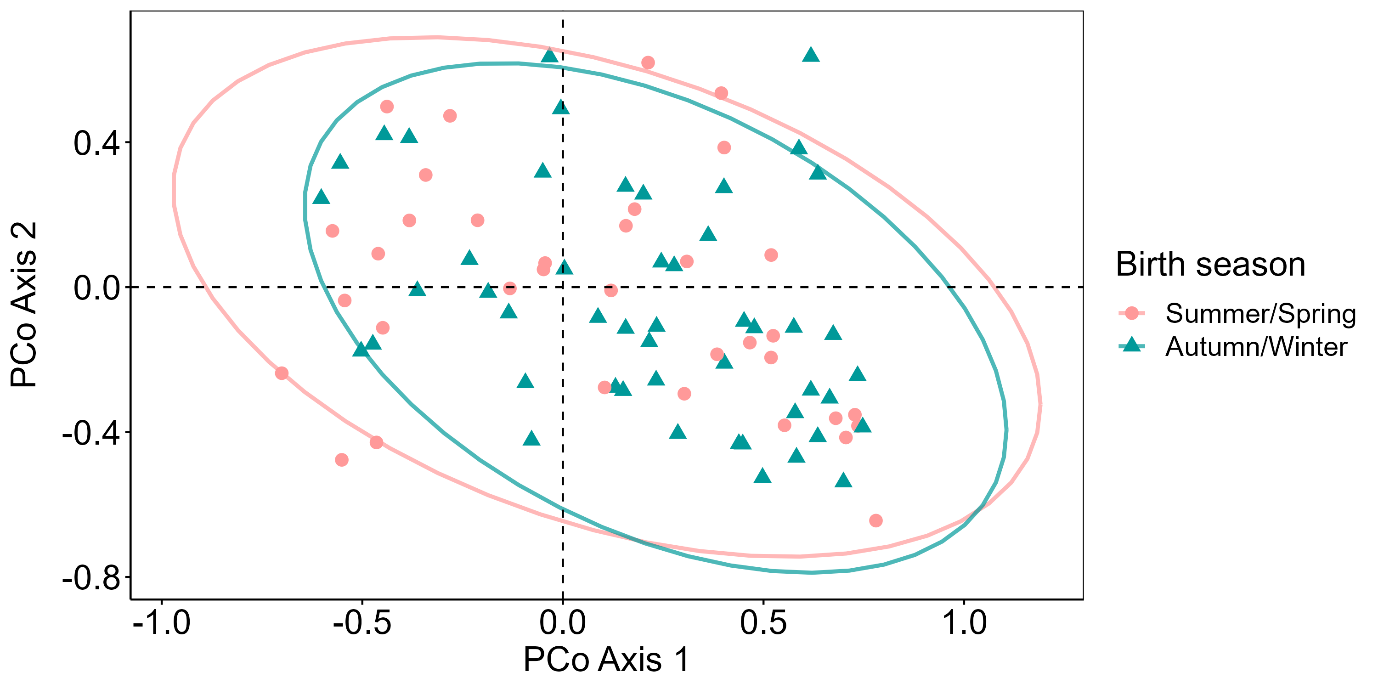
 **Supplementary Figure S12.** PCoA plot of the beta diversity of the infant oral microbiome by birth season. Points represent infants: pink circles indicate infants born in summer/spring, while blue triangles represent those born in autumn/winter. Ellipses illustrate the clustering within each group.


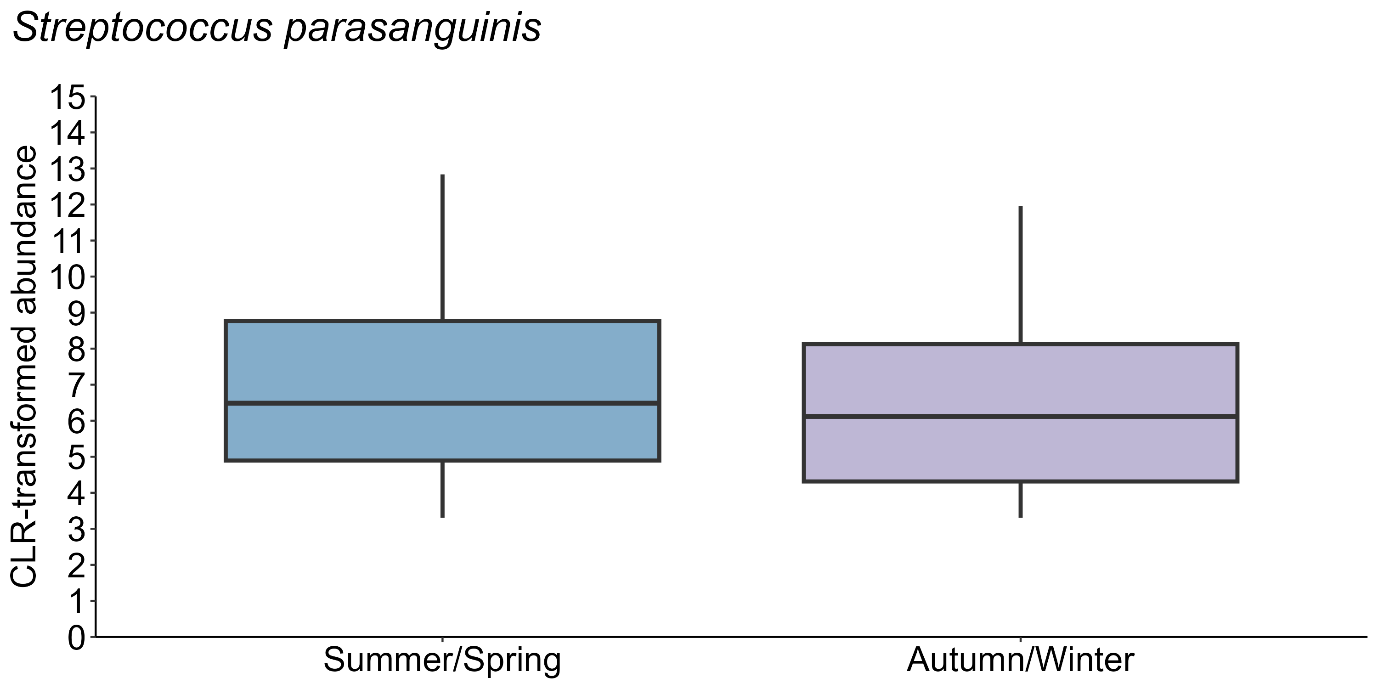


**Supplementary Figure S13.** Reduced levels of *Streptococcus parasanguinis* in the oral cavity of infants born in winter/autumn (n = 49) compared to those born in summer/spring (n = 35) (P=0.007). Data shown for the 1-month time point, with 1 month as the reference for age comparisons.
